# CRISPR/Cas9 editing in human pluripotent stem cell-cardiomyocytes highlights arrhythmias, hypocontractility, and energy depletion as potential therapeutic targets for hypertrophic cardiomyopathy

**DOI:** 10.1093/eurheartj/ehy249

**Published:** 2018-05-08

**Authors:** Diogo Mosqueira, Ingra Mannhardt, Jamie R Bhagwan, Katarzyna Lis-Slimak, Puspita Katili, Elizabeth Scott, Mustafa Hassan, Maksymilian Prondzynski, Stephen C Harmer, Andrew Tinker, James G W Smith, Lucie Carrier, Philip M Williams, Daniel Gaffney, Thomas Eschenhagen, Arne Hansen, Chris Denning

**Affiliations:** 1 Department of Stem Cell Biology, Centre of Biomolecular Sciences, University of Nottingham, UK; 2 Department of Experimental Pharmacology and Toxicology, Cardiovascular Research Center, University Medical Center Hamburg-Eppendorf, Hamburg, Germany; 3 Partner Site Hamburg/Kiel/Lübeck, DZHK (German Center for Cardiovascular Research), Hamburg, Germany; 4 The Heart Centre, William Harvey Research Institute, Barts and The London School of Medicine and Dentistry, Charterhouse Square, London, UK; 5 Molecular Therapeutics and Formulation. School of Pharmacy, University of Nottingham, UK; 6 Wellcome Trust Sanger Institute, Wellcome Genome Campus, Hinxton, Cambridge, UK

**Keywords:** Hypertrophic cardiomyopathy, Disease modeling, CRISPR/Cas9, Genome-edited human pluripotent stem cell-cardiomyocytes, R453C-βMHC

## Abstract

**Aims:**

Sarcomeric gene mutations frequently underlie hypertrophic cardiomyopathy (HCM), a prevalent and complex condition leading to left ventricle thickening and heart dysfunction. We evaluated isogenic genome-edited human pluripotent stem cell-cardiomyocytes (hPSC-CM) for their validity to model, and add clarity to, HCM.

**Methods and results:**

CRISPR/Cas9 editing produced 11 variants of the HCM-causing mutation *c.C9123T-MYH7* [(p.R453C-β-myosin heavy chain (MHC)] in 3 independent hPSC lines. Isogenic sets were differentiated to hPSC-CMs for high-throughput, non-subjective molecular and functional assessment using 12 approaches in 2D monolayers and/or 3D engineered heart tissues. Although immature, edited hPSC-CMs exhibited the main hallmarks of HCM (hypertrophy, multi-nucleation, hypertrophic marker expression, sarcomeric disarray). Functional evaluation supported the energy depletion model due to higher metabolic respiration activity, accompanied by abnormalities in calcium handling, arrhythmias, and contraction force. Partial phenotypic rescue was achieved with ranolazine but not omecamtiv mecarbil, while RNAseq highlighted potentially novel molecular targets.

**Conclusion:**

Our holistic and comprehensive approach showed that energy depletion affected core cardiomyocyte functionality. The engineered R453C-βMHC-mutation triggered compensatory responses in hPSC-CMs, causing increased ATP production and αMHC to energy-efficient βMHC switching. We showed that pharmacological rescue of arrhythmias was possible, while *MHY7*: *MYH6* and mutant: wild-type *MYH7* ratios may be diagnostic, and previously undescribed lncRNAs and gene modifiers are suggestive of new mechanisms.

**Figure:**
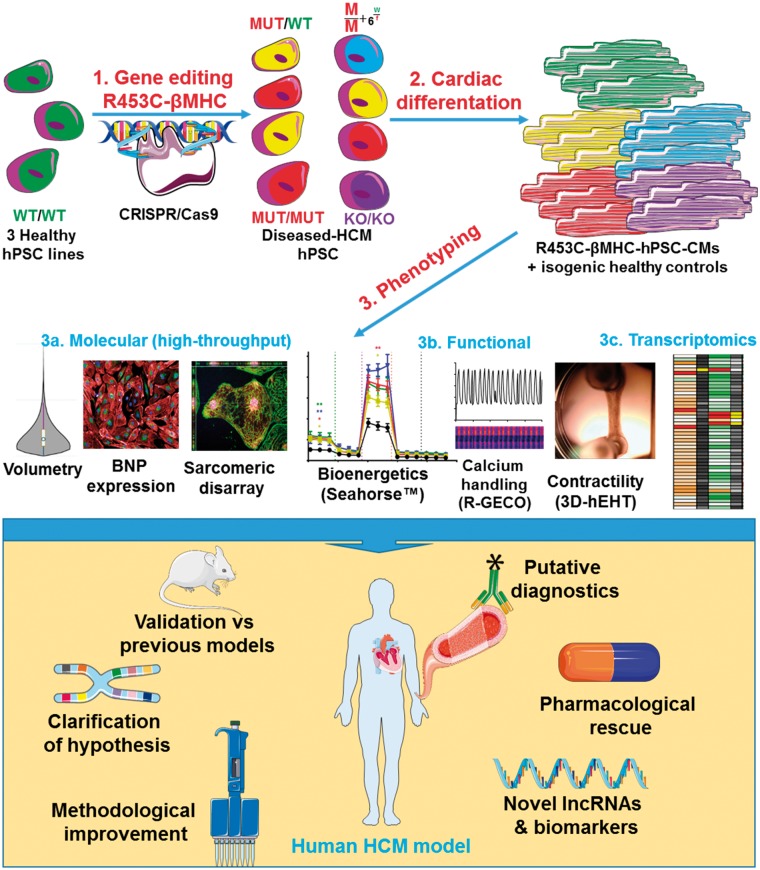

**See page 3893 for the editorial comment on this article (doi: 10.1093/eurheartj/ehy388)** 

**Translational perspectives**

Affecting 1:500 people, hypertrophic cardiomyopathy (HCM) is a complex cardiovascular disease of high clinical heterogeneity, which can cause heart failure. Therapies have remained static, often involving invasive surgery. Differences in physiology and subtleties in gene expression confound use of animal models and heterologous systems, while usable human material is scarce. To create a new, high-precision model of HCM, CRISPR/Cas9 engineering produced isogenic β-MHC variants in human pluripotent stem cell-derived cardiomyocytes (hPSC-CMs). Unrivalled molecular and function phenotyping validated HCM hPSC-CM utility, whilst adding clarity to current working hypotheses, showing potential for pharmacological rescue of arrhythmias, suggesting putative diagnostics, and pointing towards new targets for mechanistic understanding and therapeutics.

## Introduction

Affecting 1:500 individuals, HCM is the most prevalent cardiac disease,^1^^,^^2^ often leading to sudden cardiac death at a young age (48 ± 19 years).^3^ Clinical spectrum varies from asymptomatic to severe cardiac dysfunction.^4^^,^^5^ Half of HCM patients bear mutations in one or more of >20 sarcomeric genes, leading to variable penetrance of the disease.^6^ This implies influence of factors beyond the single pathogenic mutation, such as genetic background^7^ and environmental modifiers.^8^ Genetic heterogeneity causes phenotypic variability, with cellular mechanisms including (i) hypertrophy, (ii) foetal gene programme initiation, (iii) energy perturbation, (iv) fibrosis, (v) contractile dysfunction, and (vi) impaired calcium cycling.^9^

Among the sarcomeric genes mutated in patients, *MYH7* is prevalent (20–50% of genotyped cases).^10^*MYH7* encodes beta myosin heavy chain (β-MHC), responsible for regulating actin–myosin interaction, hence cardiomyocyte contraction and ultimately cardiac function.^11^ Despite clinical and phenotypic heterogeneity, *MYH7* mutations are associated with more severe forms of hypertrophy relative to when other sarcomeric genes are altered. This includes higher frequencies of ventricular tachycardia, greater disease penetrance, higher risk of sudden cardiac death, and earlier onset.^12^^,^^13^

Disease modelling of HCM using hPSC-CMs offers a pathophysiologically relevant approach to dissect the mechanics of disease and identify new targets for pharmacological intervention.^14^ While previous *MYH7*-HCM animal models provided insight into the disease,^15^^,^^16^ data may be misinterpreted due to species differences. Most HCM hPSC-CM modelling studies have focused on limited features^17^ and/or lacked isogenic controls.^18^^,^^19^ This confounds understanding because impact of genetic background on phenotype can exceed that caused by the pathogenic mutation.^20^ The only exception is an interesting, but limited, preview of the potential utility of isogenic lines in dilated cardiomyopathy via correction of a phospholamban R14del mutation patient-specific hiPSC-CMs. Impaired cardiac contractility was restored in corrected 3D engineered cardiac tissue, although this was the only phenotype assessed.^21^

We created a comprehensive hPSC-based model of HCM via Clustered Regularly Interspaced Short Palindromic Repeats (CRISPR/Cas9) editing to make a *c.C9123T* substitution in *MYH7*, corresponding to a pathogenic protein change, p.R453C-βMHC. This included homozygous variants, not previously been reported for any HCM mutation. Extensive molecular and functional evaluation of isogenic hPSC-CMs phenocopied the main hallmarks of hypertrophy, showing a general association between mutation load and level of phenotypic perturbation. Key outcomes included partial phenotypic rescue of arrhythmias with ranolazine, putative diagnostics via ratiometric gene analysis, and RNAseq highlighting a potential role of several long non-coding RNAs (lncRNA) and gene modifiers. This will guide future work on mechanistic understanding, management, and treatment of HCM.

## Methods

See Supplementary material online for details.

## Results

### CRISPR/Cas9 engineering and characterization of *MYH7* variants

**Figure 1:**
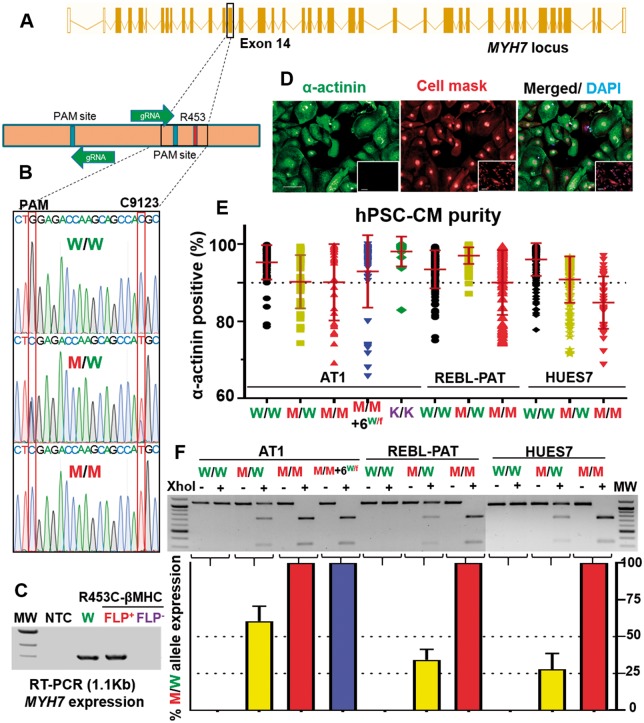
CRISPR/Cas9 engineering of *MYH7*. (*A*) Schema of *MYH7* highlighting target and Protospacer Adjacent Motif (PAM) for nickase CRISPR/Cas9 editing. (*B*) Genotyping of *MYH7* in human pluripotent stem cells, introducing *c.C9123T* and showing a silent mutation (*TGG* PAM to *TCG*). (*C*) RT-PCR showed selection cassette causes an *MYH7* knockout; Flippase-mediated excision restores expression. (*D*) Immunostaining of cardiac α-actinin human pluripotent stem cell-cardiomyocytes and fibroblast controls (inset); cell mask and DAPI as counterstains. Bar = 100 μm. (*E*) Cardiomyocyte differentiation purity >90% α-actinin + cells; *n* = 8; Bar = 100 µm. (*F*) RFLP of *MYH7*, with ratiometric densitometry of MUT: wild-type (*n* = 4). FLP, Flippase; MW, molecular weight; NTC, non-template control. Data, mean ± SD.

Since the c.C*9123T-MYH7* mutation associates with HCM pathophysiology, we coupled a dual gRNA/Cas9-nickase/CRISPR targeting strategy with subsequent flippase-mediated cassette excision to produce 9 polymorphic variants (*Figures 1A* and *B*; Supplementary material online, *Figure S1A–E* and *Tables S1–S3*). For each of 3 hPSC lines (AT1-hiPSC, REBL-PAT-hiPSC, HUES7-hESC), isogenic sets included *9123-MYH7* parental (C/C wild-type, termed **W**T/**W**T), heterozygote (T/C, mutant - **M**UT/**W**T), and homozygote (T/T, **M**UT/**M**UT). Two additional AT1-hiPSC isogenics were included (*Figure 1C*; Supplementary material online, *Figure S1F*): Line ‘homozygote *+* *MYH6^WT/fs^*’ was mutant (T/T) for *9123-MYH7* with an off-target **f**rameshift event in one allele of the homologous gene, *MYH6.* With the exception of this line, no other off-target events were detected; Line *MYH7* ‘Knockout’ (*MYH7-***K**O) contained the selection cassette, which disrupted RNA and protein expression; flippase-mediated cassette excision restored expression (*Figure 1C*). This isogenic approach minimises the genetic and epigenetic variability seen between individuals and allows impact of the R453C-βMHC mutation to be isolated.

Mono- and bi-allelic targeting frequency across the 11 lines was 16.3–25.5% and 2.6–7.1%, respectively (Supplementary material online, *Figure S1D, E*). Isogenics maintained pluripotency, including high efficiency cardiac differentiation; only cultures of ≥85% α-actinin positive were used (*Figures 1D, E*; Supplementary material online, *S2A–D*). β-Myosin heavy chain was expressed, except in *MYH7*-KO (Supplementary material online, *Figure S2D*). Pertinent to HCM, a ventricular cardiac subtype was predominant (Supplementary material online, *Figure S3*)^22–24^ By immunostaining, 91.3 ± 6.4% of cardiomyocytes were MLC2v^+^. Functional data from patch clamp of single cells or CellOPTIQ-based optical imaging of synchronous monolayers (Supplementary material online, *Figure S3C, D*) showed ∼60 to 90% ventricular-like morphologies using two separate analysis approaches (APD_90_/APD_50_)^23^ and (APD_80_-APD_70_)/APD_40_-APD_30_).^24^

**Figure 2:**
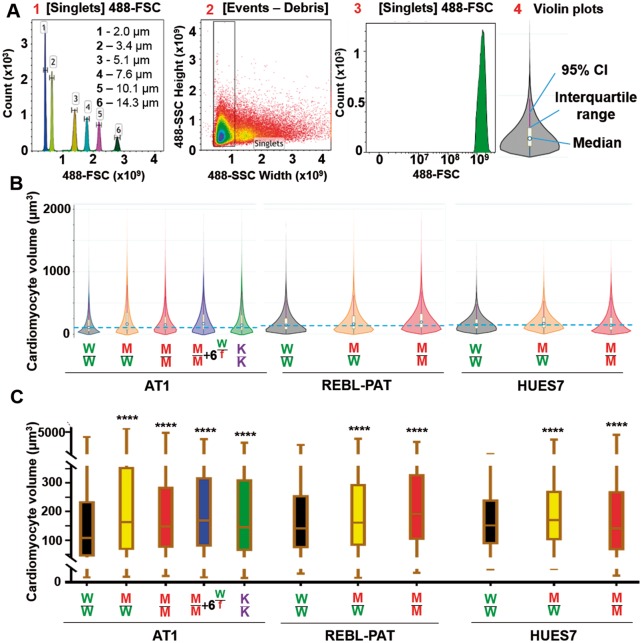
Flow cytometry calculation of human pluripotent stem cell-cardiomyocyte hypertrophy. (*A*) Calibration of forward scatter (FSC) from predefined beads sizes allowed human pluripotent stem cell-cardiomyocyte size quantification. (*B*) Violin plots (25 000 cells/sample) show volume of AT1 (*n* = 9), REBL-PAT (*n* = 6), and HUES7 (*n* = 3) lines. Dotted blue line indicates median of volume of isogenic controls. (*C*) Box/whiskers plots show interquartile range of volume, and highlights higher median and first quartile metrics in gene-edited lines. *P*-values, one-way ANOVA test + Dunnett’s correction; ****Significance all <0.0001.

**Figure 3:**
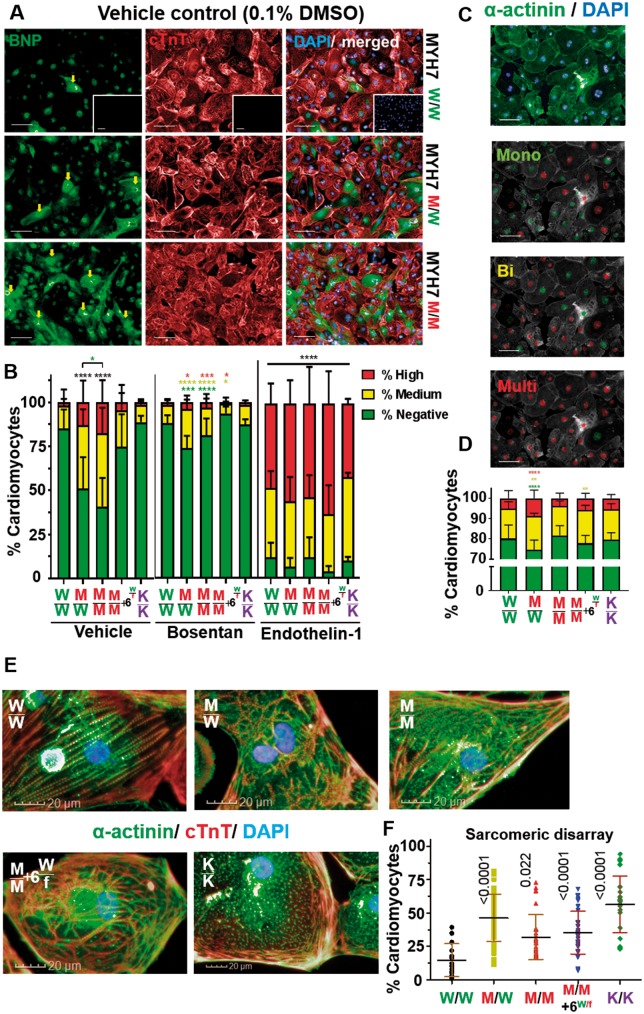
Phenotyping hypertrophic cardiomyopathy using high-content imaging. (*A*) brain natriuretic peptide/cardiac Troponin T (cTnT)/DAPI-immunostained human pluripotent stem cell-cardiomyocytes (fibroblasts, negative control; inset). Arrows indicate brain natriuretic peptide positive cells. Bar = 100 μm. (*B*) Percentage of human pluripotent stem cell-cardiomyocytes with negative, medium, or high brain natriuretic peptide expression were binned using predetermined thresholds. Bosentan treatment (100 nM) rescued brain natriuretic peptide of mutant lines to isogenic control levels; Endothelin-1 treatment (10 nM) maximized brain natriuretic peptide expression (*n* = 6). (*C*) Images of α-actinin/DAPI-immunostained human pluripotent stem cell-cardiomyocytes analysed by the algorithm to distinguish between mono-, bi-, and multi-nucleation (Bar = 100 μm), with (*D*) displaying quantification (*n* = 8). (*E*) Images of human pluripotent stem cell-cardiomyocytes immunostained for sarcomeric banding (Bar = 20 μm), with (*F*) displaying quantification (*n* = 4). Data, mean ± SD. *P*-values shown. One-way ANOVA test + Dunnett’s correction compared mutant lines vs. their isogenic control. Student’s *t*-tests, treated vs. vehicle control (*P < 0.05; **P < 0.01; ***P < 0.005; ****P < 0.0001, absolute numbers in Supplementary material online, *Table S4*). Colour-coded by inter-compared category (black asterisks apply to all categories).

**Figure 4:**
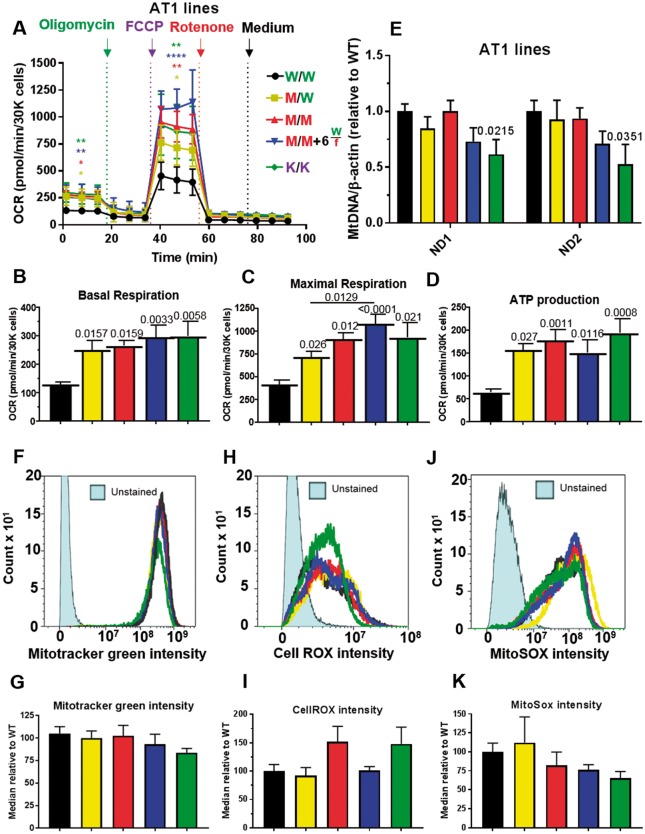
Cardiac bioenergetics analysis of AT1-hypertrophic cardiomyopathy lines. (*A*) Mitochondrial respiration profile of AT1 R453C-β-myosin heavy chain human pluripotent stem cell-cardiomyocytes using the Seahorse platform quantified, (*B*) basal respiration, (*C*) maximal respiration, (*D*) ATP production (*n* = 5). (*E*) qPCR analysis of mitochondrial (ND1-2): nuclear (β-actin) DNA ratio (*n* = 3). Histograms of human pluripotent stem cell-cardiomyocytes labelled with (*F*) mitotracker, (*H*) CellROX, and (*J*) MitoSOX obtained by flow cytometry, with corresponding quantification (*G, I, K*), *n* = 5. Data, mean ± SD. *P*-values are shown. One-way ANOVA test + Dunnett’s correction compared mutant lines vs. isogenic control. Student’s *t*-tests, treated vs. vehicle control colour-coded by inter-compared category.

**Figure 5:**
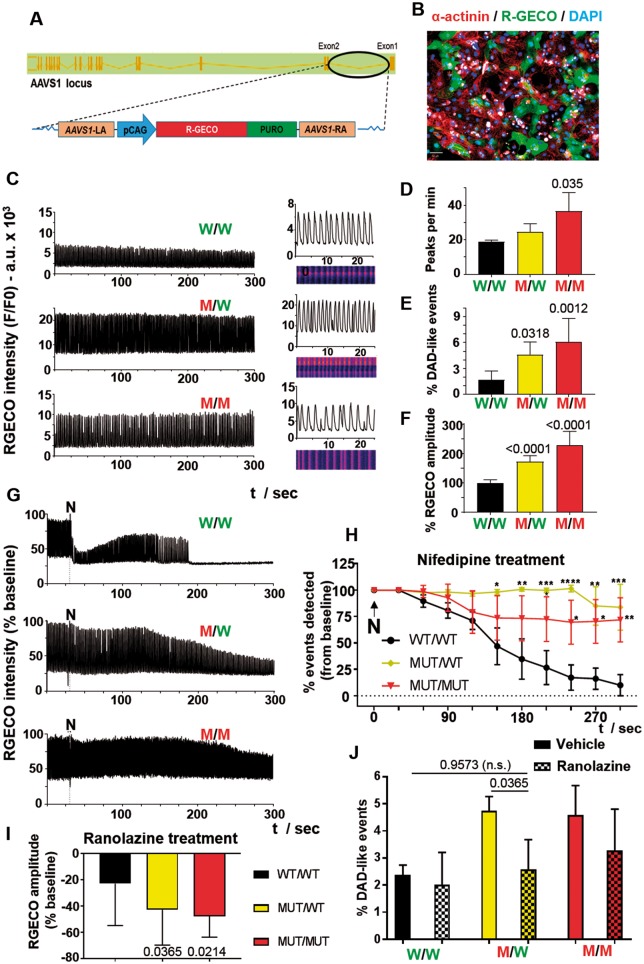
Calcium handling in hypertrophic cardiomyopathy lines by optical mapping. (*A*) A red-genetically encoded calcium indicator expression cassette was engineered into *AAVS1* of *MYH7*-mutant human pluripotent stem cell via nickase CRISPR/Cas9 editing. (*B*) α-Actinin/red-genetically encoded calcium indicator/DAPI-immunostained human pluripotent stem cell-cardiomyocytes post-gene editing demonstrates expression of the red-genetically encoded calcium indicator calcium sensor (Bar = 50 μm). (*C*) Optical mapping of R453C-β-myosin heavy chain REBL-PAT cardiomyocytes (*n* = 10) enabled quantification of (*D*) beat rate, (*E*) DAD-like abnormal events, and (*F*) signal amplitude (correlated with increased systolic calcium peak). Treatment of gene-edited lines with 1 μM nifedipine (*n* = 5) is in (*G, H*). Impact of 1 μM ranolazine (*n* = 6) on red-genetically encoded calcium indicator signal amplitude in (*I*) and frequency of DAD-like events in (*J*). Data, mean ± SD. *P*-values, one-way ANOVA test + Dunnett’s correction.

**Figure 6:**
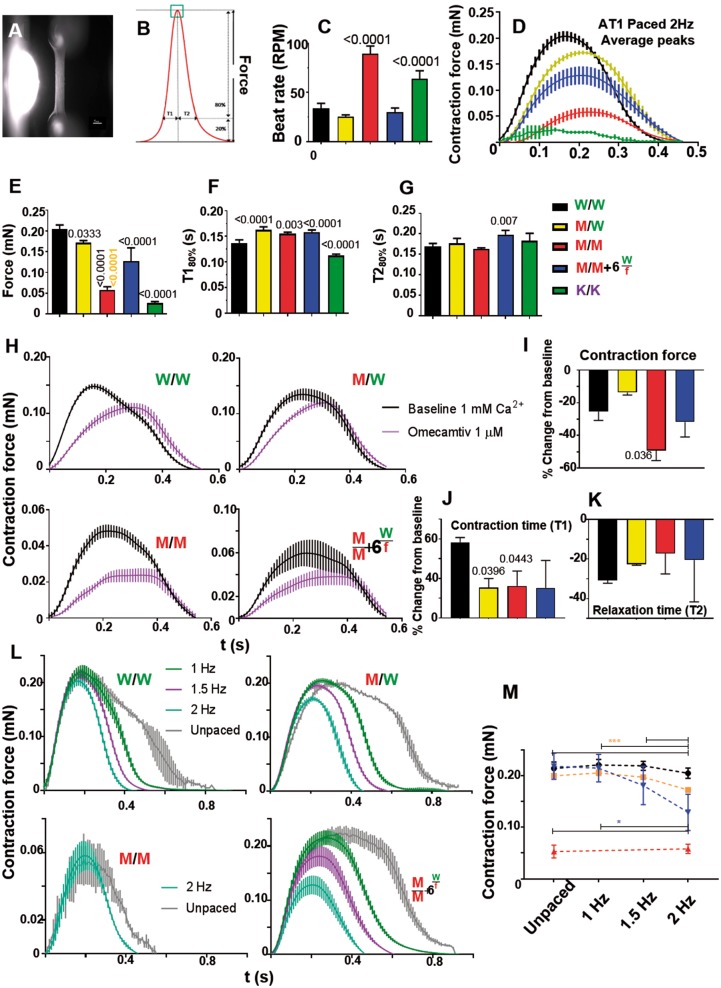
Contractile force analysis in AT1-human engineered heart tissues. (*A*) Fibrin-based AT1-human engineered heart tissue attached to silicone posts (Bar = 1 mm). (*B*) Schematic contraction peak showing parameters analysed, providing data on (*C*) spontaneous beat rate (*n* = 8). Electrically paced engineered heart tissues produced average contraction peaks (*D*), quantified for (*E*) contraction force, (*F*) contraction time, and (*G*) relaxation time (*n* = 4). (*H*) 2 Hz electrically paced AT1-engineered heart tissues with or without omecamtiv mecarbil treatment produced average contraction peaks, quantified for (*I*) contraction force, (*J*) contraction time, and (*K*) relaxation time (*n* = 3). (*L*) Force-frequency relationship in *MYH7*-mutant AT1-engineered heart tissues was assessed and quantified in (*M*). Fast spontaneous beat rate of homozygous R453C-β-myosin heavy chain mutant meant only 2 Hz pacing was possible. (*n* = 4). Data, mean ± SD. *P*-values, one-way ANOVA test + Dunnett’s correction.

Operator bias was reduced wherever possible by high-content and/or high-throughput approaches (*Figures 1–4, *and*7*; Supplementary material online, *Figures S3, S4, S6, S9*), and/or blinding the experimenter to genotype (*Figures 5*and*6*, Supplementary material online, *S7* and *S8*). While single cell patch clamp can assess genotype-cardiac subtype correlations,^25^ we avoided this technique due to low technical throughput, selection bias, influence of cell density,^26^ and loss of electrical syncytium. Dispersal of cardiomyocytes from rabbit ventricular wedges causes high levels of single cell electrophysiological heterogeneity (personal communication, Godfrey Smith, Glasgow), an effect also seen in dispersed hPSC-CMs (Supplementary material online, *Figure S3C, D*).^27^

### Molecular assessment of β-MHC mutant hPSC-CMs

We evaluated molecular characteristics of hPSC-CMs from *MYH7* isogenic sets to determine which features of HCM were replicated and clarify outstanding or controversial questions. Unequal expression of mutant and wild-type *MYH7* alleles was reported in ventricular biopsies from HCM patients.^28^ We used XhoI-based restriction fragment length polymorphism (RFLP) analysis on isogenic hPSC-CMs (*Figure 1F*). Real time- Polymerase Chain Reaction (RT-PCR) products from WT/WT lines were refractory to XhoI digestion, while MUT/MUT products were digested to 2 bands. In heterozygotes, the ratio of MUT: WT alleles ranged from 25% to 35% (HUES7, REBL-PAT) to 60% (AT1), confirming unequal expression but also variation between lines.

Increased cardiomyocyte size is archetypical of HCM.^9^ We developed a novel high-throughput (25 000 cells/sample), non-subjective and statistically powerful flow cytometry method to calculate hPSC-CM volume, whilst avoiding pitfalls of 2D analysis, including influence of cell area by substrate properties,^29^ time in culture,^30^ and serum supplementation.^31^ Forward scatter of calibration spheres generated a standard curve, enabling calculation of hPSC-CM size (*Figure 2A*). Relative to WT/WT, median volume of edited lines increased (12–51%), showing βMHC-R453C mutations cause hypertrophy in hPSC-CMs (*Figure 2B, C*).

Brain natriuretic peptide (BNP) is elevated >100-fold in plasma from HCM patients.^32^ We adapted high-content imaging methods^33^ to assess ≥60 000 cells/sample for BNP expression in hPSC-CMs (*Figure 3A*). Data were binned into high, medium, or low/negative populations using predetermined empirical thresholds (*Figure 3B*; Supplementary material online, *S4A–C*). There was general association between percentages of hPSC-CMs expressing medium/high levels of BNP and increasing mutation load. Relative to WT/WT, WT/MUT, and MUT/MUT were ∼1.5 to 3.3 and ∼1.9- to 4-fold higher, respectively, with consistency across the three hPSC lines. Surprisingly, homozygote *+* *MYH6^WT/fs^* line and *MYH7*-KO did not show increased BNP expression, suggesting this phenotype could be specific to the βMHC-453 arginine to cysteine substitution. Expression of BNP could also be blocked by treatment with endothelin-1 (ET1) receptor antagonist, bosentan,^34^ or exaggerated by the known hypertrophic stimulator, ET1.^33^ Response of edited hPSC-CMs generally differed significantly to WT/WT (*Figure 3B*; Supplementary material online, *S4A–E*).

Multi-nucleation is a controversial feature of HCM in human hearts. While some reports found bi-nucleated cardiomyocytes increased in hypertrophied hearts (28.7% vs. 13.5% for healthy),^35^ others observed no differences.^36^ We performed dual α-actinin/4′,6-diamidino-2-phenylindole (DAPI) staining of hPSC-CMs from isogenic sets and developed a high-content imaging algorithm (40 000 cells/line) to distinguish mono-, bi-, and multi-nucleation (*Figure 3C, D*; Supplementary material online, *Figure S4F–G*). Strikingly, across all isogenic sets, WT/MUT showed a significant increase in bi- and multi-nucleation (1.9–3.6% and 1.1–1.9%) relative to WT/WT, suggesting an association with ratio of healthy: mutant βMHC proteins.

We explored sarcomeric structure, since myofibrillar disarray was reported in HCM hiPSC-CM lines^17^ and associates with cardiomyocyte dysfunction.^37^ To overcome commonly reported subjective methods,^38^^,^^39^ we developed a novel high-content, machine learning approach to identify morphology and texture of ≥40 000 cells/line (*Figure 3E*; Supplementary material online, *Figure S4H, I*). While disarrayed sarcomeres were detected in 14.0 ± 2.6% of WT/WT hPSC-CMs, significant increases were observed in all mutant lines, with a four-fold increase (to 56.4 ± 4.3%) in *MYH7*-KO (*Figure 3F*). Thus, disruption trended as a function of mutation load in R453C-βMHC hPSC-CMs.

Since βMHC is known to interact with multiple proteins in, or associated with, the sarcomere, we explored the impact of the R453C mutation. Previously, elegant experiments using yeast-two-hybrids showed that mutations in cMyBP-C abolished interaction with βMHC S2 (tail) domains.^40^ However, in yeast, expression of full length proteins of >1500 amino acids, such as βMHC is challenging.^41^ As an alternative, we used *in silico* modelling. Current structural homology models^42–45^ indicate the head domain (S1) of βMHC interacts with: itself (via S1–S2); cMyBP-C; actin; ATP.

R453 is located between the HCM loop and Switch-2 of βMHC S1 and interacts with the proximal S2 region when folded back. The change to cysteine is predicted to interfere with this S1–S2 βMHC interaction, by disrupting the hydrogen bond established between R453 and Q882 (Supplementary material online, *Figure S5A–C*). R453 is located close to the interface of cMyBP-C (Supplementary material online, *Figure S5D*) and, in molecular dynamics simulations, can form contacts with its C1 domain (Supplementary material online, *Figure S5E*). Contrastingly, R453 is located away from the predicted interface of S1 with actin (Supplementary material online, *Figure S5F*) and ATP-binding region (Supplementary material online, *Figure S5G*), so the mutation is not predicted to interfere directly with these interactions.^45^ However, targeted molecular dynamics simulations have shown R453C to cause changes in the flexibility of the loop between the motor domain and the actin binding site.^46^

### Mitochondrial respiration rates are perturbed in βMHC mutant hPSC-CMs

Although controversial, one working hypothesis states that mutant proteins within the sarcomere cause inefficient sarcomeric ATP utilization, energy depletion, increased oxygen consumption, and cardiac dysfunction.^47^^,^^48^ To determine whether or not our model supported this hypothesis, we analysed the isogenic hPSC-CMs with the Seahorse platform (*Figure 4A*; Supplementary material online, *Figure S6A*). This profiled oxygen consumption rates (OCR) during sequential addition of electron transport chain inhibitors, enabling calculation of basal and maximal respiration rates. The Seahorse also measures ATP production in a manner that correlates to outputs from other direct approaches, such as Luciferase ATP assay.^49^ There was a positive association between these parameters and mutation load, such that *MYH7*-KO and homozygote + *MYH6*^WT/fs^ had the highest values followed in order by MUT/MUT, WT/MUT, and WT/WT (*Figure 4B–D*; Supplementary material online, *Figure S6B–D*). This was most striking in AT1-hiPSC-CMs, where basal respiration, maximal respiration and ATP production increased by ∼3-, 2.75-, and ∼3-fold, respectively.

Surprisingly, these changes were not due to greater mitochondrial content, since mitochondrial: nuclear DNA ratio^50^ showed little difference (*Figure 4E*; Supplementary material online, *Figure S6E*). This was supported by flow cytometry using Mitotracker^®^ (*Figure 4F, G*; Supplementary material online, *Figure S6F, G*), which reports on mitochondrial content and function.^51^ We speculated that similar mitochondria content in the mutant lines necessitated harder work to meet the energy demands, hence lead to increased reactive oxygen species (ROS) and cell stress. However, flow cytometry calculation of total cell ROS or mitochondrial-specific ROS showed little interline difference (*Figure 4H–K*; Supplementary material online, *S6H–K*). Thus, the isogenic lines supported the energy depletion model but did not suggest any dramatic increases in cell stress via ROS production, at least under these test conditions.

### βMHC mutant hPSC-CMs show altered calcium handling

Calcium handling is central in excitation–contraction regulation, hence development of HCM. We used nickase CRISPR/Cas9 to knock-in a red genetically encoded calcium indicator (R-GECO1) expression cassette into the safe *AAVS1* locus of isogenic REBL-PAT-hiPSC-CM trio (*Figure 5A, B*; Supplementary material online, *Figure S7A–D*). Calcium imaging was analysed by confocal line scans (*Figure 5C–F*). Relative to WT/WT, there was an upward trend in beat rate (50% in MUT/MUT), frequency of delayed after depolarization (DAD)-like events interspersing the main peaks (>9-fold increase in MUT/MUT) and signal amplitude/higher systolic calcium peak (WT/MUT, ∼1.72-fold; MUT/MUT, ∼2.27-fold). Availability of cytosolic calcium to trigger beating in diseased lines for longer was corroborated by treatment of cardiomyocytes with 1 μM nifedipine, an L-type calcium channel blocker (*Figure 5G, H*).

These findings suggested that higher cytosol calcium concentrations caused DAD-like arrhythmias in mutant hPSC-CMs. To explore whether pharmacological rescue was possible, hPSC-CMs were treated with 1 μM ranolazine, which acts as an enhancer of the outward mode of sodium-calcium exchanger (NCX) by blocking late sodium currents, hence indirectly promotes Ca^2+^ efflux.^52^ This led to reduced R-GECO1 signal amplitude and frequency of DAD-like events in diseased lines, particularly WT/MUT (*Figure 5I, J*, Supplementary material online, *Figure S7E*). Thus, altered calcium handling and arrhythmogenesis were identified in R453C-βMHC lines, and partial rescue could be achieved with ranolazine.

### 3D engineering unveils hypo-contractility, negative clinotropy, and an exacerbated negative force–frequency relationship

Contraction is the fundamental purpose of the heart. Human engineered heart tissues (hEHTs) directly measure contraction force by partially recapitulating the 3D architecture of cardiac tissue by imposing the auxotonic tension present *in vivo.*^53^^,^^54^ We produced hEHTs from AT1 and REBL-PAT isogenics (*Figure 6A, B*), wherein cardiomyocytes exhibited excellent alignment (Supplementary material online, *Figure S8A*). Beat rate was fast in MUT/MUT and *MYH7*-KO (*Figure 6C*). Analysis of force under 2 Hz pacing (*Figure 6D–G*; Supplementary material online, *Figure S8C–F*), contraction time (T1_80%_), and relaxation time (T2_80%_) produced trends similar to the 2D assays; mutation load associated with poorer functional output. There was a predominance of hypocontractility and negative clinotropy (increased T1_80%_), although little change in T2_80%_. Contraction in *MYH7*-KO was so compromised (∼ten-fold lower force than WT/WT), measurements were almost impossible (*Figure 6D, E*).

We attempted pharmacological rescue of reduced contraction force and increased contraction time in hEHTs formed from mutant hPSC-CMs. Omecamtiv mecarbil is a cardiac myosin activator that acts by prolonging the actin–myosin interaction state, thereby extending systolic ejection time and increasing cardiac contractility.^55^ AT1- and REBL-PAT-hEHTs treated with 1 µM omecamtiv mecarbil enhanced negative clinotropy but, unexpectedly, decreased contractile force, apparently exacerbating impact of the 453-βMHC mutation (*Figure 6H–K*; Supplementary material online, *Figure S8G–J*).

The inability of HCM-afflicted hearts to produce more force during exercise-induced increases in beat rate contributes to sudden cardiac death.^56^ We simulated this scenario in hEHTs. In AT1-hiPSC-CM-EHTs, force declined as a function of pacing frequency in WT/MUT and homozygous + *MYH6*^WT/fs^ lines (*Figure 6L, M*). Measurement was not possible in AT1-MUT/MUT due to the high spontaneous beat rate. Unlike AT1-EHTs, decline in force was not seen during stepped pacing of hEHTs formed from REBL-PAT-hiPSC-CMs (Supplementary material online, *Figure S8K*). This may be due to their high baseline beat rates (∼1.5 to 2 Hz) and/or low intrinsic force production (0.04–0.06 mN) (Supplementary material online, *Figure S8B*), or may relate to the higher expression of the mutant allele in AT1 vs. REBL-PAT heterozygotes (60% vs. 35%, respectively) (*Figure 1F*).

### RNA-seq highlights lncRNAs as potential therapeutic candidates for HCM

**Figure 7:**
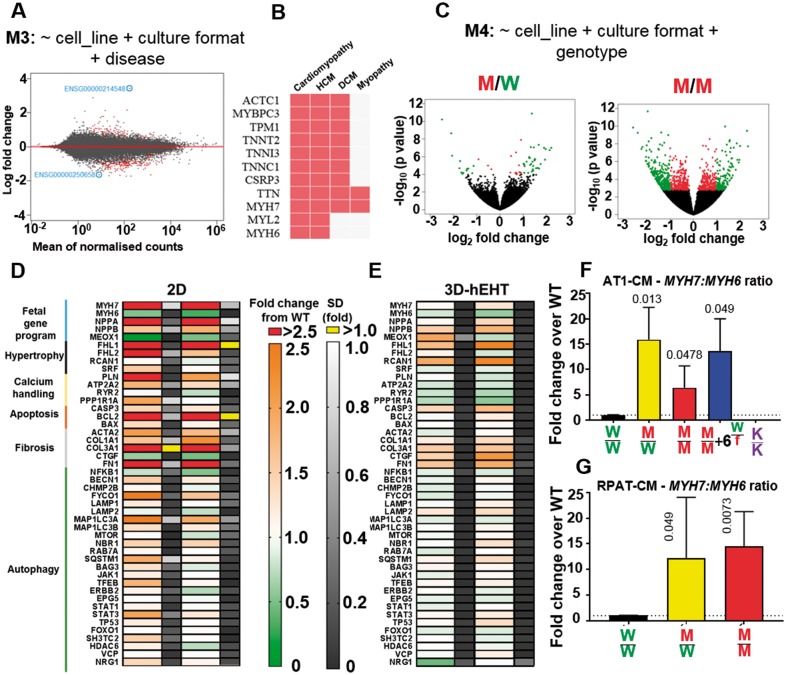
Transcriptomics analysis of hypertrophic cardiomyopathy lines. (*A*) MA plot of RNA-sequencing model developed showing differentially expressed genes (766, false discovery rate < 0.1) between wild-type and diseased (*MYH7*-mutant) conditions, using cell line and culture format as controlling factors (*n* = 3). (*B*) Genes identified were enriched for cardiomyopathy Online Mendelian Inheritance in Man (OMIM) disease. (*C*) Volcano plots after refinement enabled distinction between diseased states by considering the genotype showed increasing number of differentially expressed genes with mutation load (*P* < 0.05, red; log_2_ fold > 1 change, green). Fold ± SD changes in the expression of genes involved in archetypal hypertrophic cardiomyopathy pathways in the *MYH7*-mutant REBL-PAT-cardiomyocytes s relative to wild-type in (*D*) 2D cultures and (*E*) 3D-engineered heart tissues. q-PCR analysis of *MYH7/MYH6* expression ratios in gene-edited lines in 2D cultures normalized to wild-type in (*F*) AT1 and (*G*) REBL-PAT lines (*n* = 4). Data, mean ± SD. *P*-values, one-way ANOVA test + Dunnett’s correction.

To provide new insight into HCM, we used global transcriptome analysis of isogenic hPSC-CM cultured as 2D monolayers (AT1, REBL-PAT, HUES7) and hEHTs in 3D (AT1, REBL-PAT). Principal component analysis (PCA) showed that hPSC culture format and cell line origin were the main variance factors (Supplementary material online, *Figure S9A*), reinforcing the importance of isogenic controls to model disease.^20^ Analysis from all variants in 2D and 3D identified 290 differentially expressed genes [<10% false discovery rate (FDR) Supplementary material online, *Figure S9B, C*]. Two layers of refinement were applied: first, correction for cell line of origin and culture format returned 766 genes (FDR < 0.1; *Figure 7A*). These included loci associated with several cardiomyopathies (*Figure 7B*, Supplementary material online, *Figure S9D*), but also previously unidentified lncRNAs as top hits (2- to 8-fold change); Second, sub-classifying genotypes within the diseased category into volcano plots showed number of differentially expressed genes associated with increasing mutation load (*Figure 7C*), following the trend of the phenotypic assays.

### Focused transcriptomics reveals core pathways triggered by HCM

Focused analysis of 2D and 3D samples for AT1 and REBL-PAT isogenic sets via a ∼50 genes nanoString RNA chip (*Figure 7D, E*; Supplementary material online, *S9F–G*) enabled querying of genes involved in (i) foetal gene programme, (ii) hypertrophy, (iii) calcium handling, (iv) apoptosis, (v) fibrosis, and (vi) autophagy. Data from 2D hPSC-CMs identified increased expression of genes involved in the Foetal programme (*NPPA/B*, validating BNP data), hypertrophic responses (*FHL1/2*), apoptosis (*CASP3*), and fibrosis (*FN1*). While changes were sustained in 3D (mainly fibrosis and hypertrophy), certain opposing trends were seen, corroborating conclusions from RNAseq data on the importance of culture format. In 3D, decreases in the genes involved in calcium handling machinery were found, while changes in apoptosis and autophagy were less pronounced.

From the transcriptional data, we noted changes in expression of *MYH7* and *MYH6*. These were confirmed by qRT-PCR analysis, showing ∼5- to 15-fold increases in the *MYH7*:*MYH6* ratio in the diseased lines across all three isogenic groups (*Figure 7F, G*; Supplementary material online, *Figure S9E*). This is consistent with the 3.5-fold change in *MYH7*:*MYH6* ratio caused by hypertrophy of human hearts.^57^ These observations suggest a compensatory feedback loop, whereby sarcomere inefficiency downregulates the ‘energy hungry’ fast αMHC isoform in favour of the normally ‘energy-efficient’ βMHC isoform. Altogether, transcriptomic analyses highlighted foetal gene programme initiation, hypertrophic responses, and αMHC to βMHC isoform switching as the main pathways triggered in HCM.

## Discussion

New investigative tools are needed for HCM. Few pharmacological treatments exist, and the condition can necessitate surgery and/or heart transplantation.^58^ Advancements are confounded by heterogeneity, wherein reproducibility of genotype–phenotype correlations are challenging because human material is limited and frequency of the same ‘natural’ mutations within families is low, compromising statistical power.^59^ We overcame these issues by creating 11 isogenic variants in three different hPSC lines centred on a *c.C9123T-MYH7* (p.R453C-βMHC) substitution; until now, engineered homozygotes have not been reported for human-based HCM. Our data demonstrated unequivocally that a single R453C-βMHC mutation causes a severe and penetrant pathophysiology independent of genetic background.^60^

Our use of 12 different phenotyping approaches far exceeds previous studies,^21^ showing salient features of HCM were recreated in the hPSC-CMs expressing the mutant βMHC. This validation is essential since hPSC-CMs are often cited as being immature and hence not representative of the adult cardiomyocyte or intact myocardium. Our comprehensive approach, coupled with other evidence that hPSC-CMs replicate morphology,^53^ contractility,^53^ electrophysiology,^61^ signalling,^62^ and metabolism,^63^ gives confidence that the outcomes we observed *in vitro* are also relevant for HCM *in vivo*. hPSC-CM immaturity may even be advantageous by modelling early disease stages, which is particularly relevant for R453-βMHC patients who typically show an early onset of heart failure.^60^ This is when treatment is most likely to be effective, hence will be useful for further mechanistic dissection, development of diagnostics, and drug testing. A next logical step will be further refinement by generating complex tissues or organ-on-a-chip. This will require production of other cells types found in the heart, such as cardiac fibroblasts, endothelial cells, and smooth muscle cells, although robust protocols for hPSC-based differentiation of these lineages are currently at various stages of development.^64^^,^^65^

For many of the molecular assays and functional phenotyping, the level of dysfunction associated well with mutation load but sometimes differed between the three hPSC lines, mirroring HCM complexity.^59^ Closer inspection showed association with the ratio of MUT:WT *MYH7* allele expression. Heterozygote AT1 showed the most severe phenotypes, and had a MUT:WT ratio of 60%, followed by REBL-PAT (35%) and HUES7 (25%). This raises the intriguing possibility of whether this ratio could be a diagnostic predictor of severity of pathophysiology in patients.^28^^,^^66^

Isogenic sets will add clarity to the field. The impact of HCM on mitochondrial respiration is controversial. Explants of human hypertrophied hearts showed ∼two-fold higher OCR,^48^ whereas skinned muscle bundles obtained from myocardium of explanted human hearts showed no difference relative to healthy controls.^67^ These previous studies have been constrained by variability and scarceness of the material, limitations overcome by isogenic hPSC-CMs Our data on mitochondrial function/content, calcium handling and the transcriptome support the energy depletion model of HCM, which states disorganised sarcomere causes inefficient ATP usage and imposes increased energetic demands on the cardiomyocyte.^47^ This compromises the energy available to reduce cytosolic calcium levels back to baseline, precipitating arrhythmogenesis. Interestingly, stress cues, such as energy deficits, are known to trigger ploidy-activated genes, promoting the α-MHC to β-MHC isoform switch, which leads to enhanced production of ATP and allows the cardiomyocytes to be more energy-efficient.^57^^,^^68^ Our observation of cell stress via energy depletion, increased multi-nucleation (potentially leading to polyploidy) and isoform switch supports this as an underlying mechanism of R453C-βMHC mediated HCM.

The isogenic sets supported the notion that mitochondrial content in failing hearts is not increased.^69^ We expected that increased demand from the same number of mitochondria in cardiomyocytes with inefficient sarcomeres would lead to increased ROS and cell stress, as reported in mitochondrial cardiomyopathies.^70^ At least under baseline spontaneous beating, this proved incorrect. High frequency pacing (2–4 Hz) may unveil further phenotypes. While this is not possible on the Seahorse platform, the advent of optogenetics-based methods for pacing hPSC-CMs^71^ may provide a future route of enquiry.

Our data on contractility are compatible with literature investigating HCM in human cells and tissues, but contradict rodent studies. In R453C-βMHC hPSC-CMs, we observed sarcomeric disarray and hypo-contractility, despite increased metabolic demands. Force generation in R723G-βMHC human heart biopsies or E848-βMHC hPSC-CM myofibrils was 30–66% lower.^37^^,^^72^ Tension was ∼35% lower in several βMHC-mutant tissues,^73^^,^^74^ whereas tension-cost was higher in R403Q-βMHC^75^ and R403W-βMHC^76^ variants. Contrastingly, murine C2C12 myoblasts expressing recombinant R453C-βMHC showed increased force generation and reduced maximum ATPase activity.^77^ Rodent cardiomyocyte-derived EHTs bearing missense mutations in sarcomeric genes (*FHL2*,^78^*ANKRD1*,^79^ and *MYBPC3*^80^^,^^81^) showed hypercontractile phenotypes.

These differences may underscore the delicate balance between α-MHC and β-MHC expression, which is impacted on by species differences and/or transgenic overexpression. Isoform switch from β-MHC to α-MHC occurs in ventricles of mice during development,^82^ but β-MHC is always predominant in human ventricles.^83^ Hypercontractile phenotypes associated with α-MHC mutations in mice^84^^,^^85^ may cause opposing effects in the human predominant ventricular counterpart (β-MHC). This may explain why the homozygous + *MYH6*^fs/wt^ we describe showed a higher contractile force than the homozygous line that lacks the off-target *MYH6* event. This is supported the observation that multi-nucleation, BNP expression and foetal gene programme initiation were not exhibited by hPSC-CMs bearing the additional *MYH6* mutation. Subtlety in levels of α-MHC and β-MHC isoforms may also explain why only heterozygous R453C-βMHC hPSC-CMs showed increased proportion of multi-nucleated cells.

The isogenic sets of hPSC-CMs described here will be useful to evaluate new therapies, building upon current strategies.^86^ Ranolazine reduced the number of DAD-like arrhythmias in heterozygous R453C-βMHC cardiomyocytes, which is the genotype that reflects most closely the clinical situation for patients. This suggests that further evaluation of drugs that modulate calcium within this model system will be warranted. Omecamtiv mecarbil caused negative clinotropy, consistent with the known mode of action of this drug,^55^ but did not rescue the hypocontractility. We speculate this may be due to disruption of sarcomeric interactions by the R453-βMHC mutation, as predicted by *in silico* modelling (Supplementary material online, *Figure S5*), high beat rates or to lack of t-tubules, which could prevent drug-induced activation of myosin.

**Figure 8:**
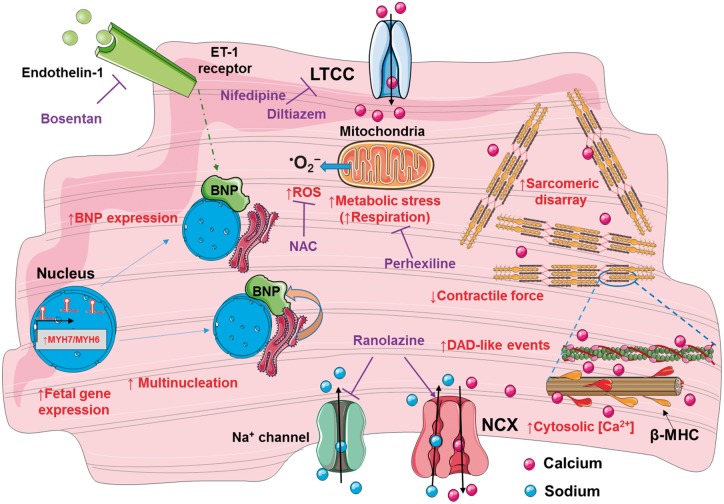
Proposed hypertrophic cardiomyopathy mechanisms. Phenotypes investigated (red) in the developed model of hypertrophic cardiomyopathy of R453C-β-myosin heavy chain human pluripotent stem cell-cardiomyocytes; putative rescue drugs (purple). LTCC, L-type calcium channels; NAC, N-acetyl-cysteine; NCX, sodium-calcium exchange pump.

In summary, we generated a scalable human model of HCM by using CRISPR/Cas9 to produce isogenic sets of *C9123T-MYH7* (R453C-βMHC) mutants in hPSC-CMs. The utility of the model was validated and now points towards routes for pharmacological rescue and diagnostics. Identification of novel lncRNAs and putative gene modifiers provide an avenue for new mechanistic and functional understanding via knockout, overexpression and pathways analysis, whereas suggesting new putative diagnostic biomarkers and targets for therapy. This model (*Figure 8*) will pave the way in evaluating single or combined drug- and/or gene-based therapeutics for HCM.

**Take home figure:**
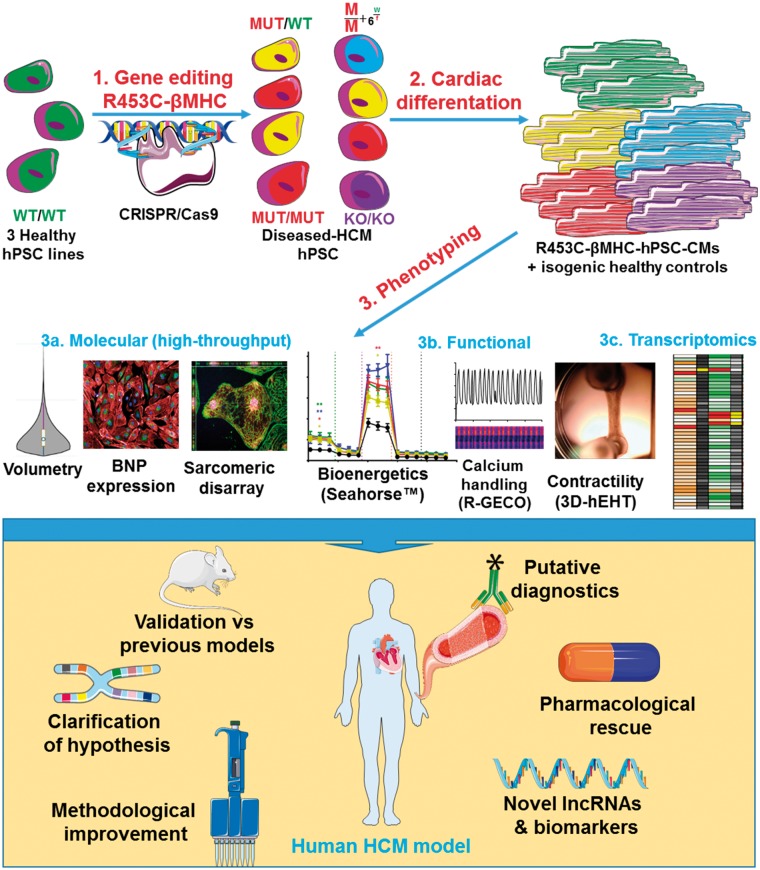
1) The R453C-betaMHC pathological change was introduced in three independent healthy hPSC lines using CRISPR/Cas9. 2) The gene edited hPSC lines were differentiated to generate isogenic sets of hPSC-cardiomyocytes. 3) Phenotyping of hPSC-CMs in terms of a) molecular, b) functional and c) transcriptomics analyses has validated the human HCM model generated, leading to new mechanistic and pharmacological understanding of the disease.

## Supplementary Material

Supplementary Data
