# Supplementary material for: CRISPR/Cas9 editing in human pluripotent stem cell-cardiomyocytes highlights arrhythmias, hypocontractility, and energy depletion as potential therapeutic targets for hypertrophic cardiomyopathy
Source: Eur Heart J. 2018 May 8;39(43):3879–92. doi: 10.1093/eurheartj/ehy249 (PMC6234851; doi:10.1093/eurheartj/ehy249)
Supplement: Supplementary Data [file ehy249_supp.zip › ehy249-suppl_data/ehy249_Supplementary_Information.docx]

**Supplementary Information**

**CRISPR/Cas9 editing in hPSC-cardiomyocytes highlights arrhythmias, hypo-contractility and energy depletion as potential therapeutic targets for HCM**

Diogo Mosqueira^1,*^, Ingra Mannhardt^2,3^, Jamie Bhagwan^1^, Katarzyna Lis-Slimak^1^, Puspita Katili^1^, Elizabeth Scott^1^, Mustafa Hassan^4^, Maksymilian Prondzynski^2,3^, Stephen C Harmer^4^, Andrew Tinker^4^, James G. W. Smith^1^, Lucie Carrier^2,3^, Philip M Williams^5^, Daniel Gaffney^6^, Thomas Eschenhagen^2,3^, Arne Hansen^2,3^ and Chris Denning^1,*^

^1^Department of Stem Cell Biology, Centre of Biomolecular Sciences, University of Nottingham, NG7 2RD, United Kingdom

^2^Department of Experimental Pharmacology and Toxicology, Cardiovascular Research Center, University Medical Center Hamburg-Eppendorf, Hamburg, Germany

^3^DZHK (German Center for Cardiovascular Research), Partner Site Hamburg/Kiel/Lübeck, Hamburg, Germany

^4^The Heart Centre, William Harvey Research Institute, Barts and The London School of Medicine and Dentistry, Charterhouse Square, London EC1M 6BQ, U.K

^5^Molecular Therapeutics and Formulation.  School of Pharmacy, University of Nottingham, NG7 2RD, United Kingdom

^6^Wellcome Trust Sanger Institute, Wellcome Genome Campus, Hinxton, Cambridge, CB10 1SA, UK

^*^Corresponding author: Tel: +44(0)115 8231236; Fax: +44(0)115 8231230;

Email: [diogo.mosqueira@nottingham.ac.uk](mailto:diogo.mosqueira@nottingham.ac.uk); chris.denning@nottingham.ac.uk

**Supplementary information contents:**

**Methods**

**9 Supplementary Figures + Legends**

**4 Supplementary Tables + Legends**

**Supplementary references**

**Methods:**

**CRISPR/Cas9 genome editing of hPSC**

**Targeting strategy and gRNA design**

Dual guide RNA/Cas9-Nickase strategy was designed to introduce the *MYH7*-C9123T SNP in three hPSC lines, encoding the R453C-β-MHC modification. The hCas9 D10A plasmid (Addgene #41816), comprising the human codon-optimized *Streptococcus pyogenes* Cas9 nuclease bearing the D10A mutation was used. Candidate sgRNAs were identified by searching for NGG PAM sites in the 150bp up- and downstream of the SNP location, using the available bioinformatics tool website http://crispr.mit.edu/, as previously described[**^1^**](#_ENREF_1). An ACCG sequence was added to the 5’ end of the gRNA sequences (in a DNA oligonucleotide), and an AAAC sequence to the 3’ end of the respective complementary sequences, annealed and cloned into BsaI-digested U6-gRNA vector backbone, using T4 DNA ligase (New England Biolabs, NEB), according to manufacturer’s instructions. Thereafter, ligated U6-gRNA vectors were transformed into in TOP10 chemically competent *E.Coli* (Thermo Fisher #C404010) and plasmid DNA was extracted by Wizard^®^ Plus SV Minipreps DNA Purification System (Promega) and analysed by BsaI-restriction digestion. The same strategy was used to engineer the RGECO-1 sensor in the AAVS1 locus of *MYH7*-C9123T hPSC.

## Targeting construct design and synthesis

The donor template targeting construct comprised the *MYH7*-C9123T SNP flanked by 1kb homology arms. It was produced by performing a 2-step PCR splice mutagenesis reaction, whereby the 2kb region was amplified using isogenic hPSC genomic DNA in two adjacent 1kb fragments, and the base changes were introduced by the primers used in **Supplementary Table 3**. Subsequently, the two 1kb fragments were annealed in the 25bp overlapping region and amplified by PCR using Phusion DNA polymerase (NEB), following manufacturer’s instructions. Cycle parameters were: 98°C for 30s for 1 cycle; 98°C for 30s, 64°C for 60-90s, 72°C for 90s for 35 cycles; 72°C for 10 min for 1 cycle. Amplified DNA was thereafter loaded in a 1% agarose (Milipore) gel stained with Ethidium Bromide (EtBr, Sigma), run at 100V for 60 min and visualized in Fujifilm LAS-4000 System & Software (GE Healthcare LifeSciences). PCR products were purified from gel using QIAquick Gel Extraction Kit (Qiagen), and Sanger Sequencing reactions were outsourced to Source BioSciences, using primers from **Supplementary Table 3**, up to 500bp up/downstream from the regions of interest. The generated 2Kb construct was subsequently cloned into pUC57 vector (Eurofins) by HindIII and SacI double digestion and ligation, with bacterial transformation and plasmid DNA purification being performed as above. Afterwards, NotI/AvrII-digested pUC57-R453C-βMHC insert was cloned into the pR6K-R1R2 backbone, using the same restriction enzymes and ligation procedure. Subsequently, the previously synthesized L1L2 FRT-Blast/Puro plasmids were recombined with the pUC57-βMHC-R453C-pR6K vector previously generated, by a Gateway LR Clonase reaction (Invitrogen #11791019), according to the manufacturer’s instructions. The final targeting constructs (pUC57- βMHC-R453C-Blast/Puro) isogenic to each hPSC line were analysed by diagnostic digestions and confirmed by Sanger Sequencing reactions, as above. All the restriction enzymes were purchased from NEB, and digestions were performed at 37°C for 2h followed by 1h at 37°C in the presence of Calf Intestinal Alkaline Phosphatase (NEB) to avoid linearized plasmid religation. Unless otherwise stated, plasmids were kindly provided by William Skarnes’ group (Wellcome Trust Sanger Institute, UK). The R-GECO1.0 CDS was cloned from the pShuttle-ChetaTC-Myc-2A-R_GECO1 (kind gift from Dr. Matthew Daniels) into the pUC57 backbone upstream of an IRES-puromycin sequence, driven by the CAG promoter and flanked with arms of homology to AAVS1.

**hPSC transfection and selection**

hPSC were seeded at approximately 15K/cm^2^ 24h prior to the transfection (as described below). 1 µg of nickase hCas9 D10A plasmid, 0.5 µg of each gRNA pU6 vector and 1 µg of the corresponding targeting vector were complexed in GeneJammer (Agilent Technologies #204132) transfection reagent, diluted 1:10 in Optimem reduced-serum medium (Invitrogen). After 15 min incubation at room temperature (RT), the transfection mixture was added to the cells and medium was changed the next day. Transfection efficiency was determined by imaging hPSC transfected with GeneJammer-GFP plasmid (pSIN-IRES-BSD-GFP) in CellaVista Cell Imager (Synentec), followed by automated estimation of GFP positive cells normalized to total cells based on positive signal area. 48h after transfection, cells were re-seeded sparsely (50 cells/ cm^2^) into MT-coated 35mm dishes (Nunc) and antibiotic treatment was performed (0.3-7.5 μg/ml Puromycin or 5-15 μg/ml Blasticydin, Invitrogen) until colonies appeared. Thereafter, mechanical passaging of single colonies was performed using a Stem Cell Cutting Tool (Vitrolife) when colonies reached an approximate 0.5–1 mm in diameter, by manually dissecting them into pieces measuring approximately 100-200μm under a stereomicroscope built into a class II safety cabinet. Individual clones were expanded and subcultured until monolayers were formed and genomic DNA was extracted using DNeasy Blood and Tissue Kit (Qiagen), following manufacturer’s instructions. Genotyping PCRs were subsequently performed with 100ng of genomic DNA, using Phusion DNA polymerase (NEB), as above (primer sequences in **Supplementary Table 3**). Afterwards, amplified DNA was loaded in a 1 % agarose gel as above, and PCR products were either purified using a PCR Purification Kit (Qiagen) or directly from gel followed by Sanger Sequencing reactions, as above. Upon confirmation of positive targeting (introduction of C9123T-*MYH7* SNP) *MYH7*-mutant hPSC lines were seeded and transfected with 3ug of pCAGGS_FlpO plasmid (wherein Blasticydin or Puromycin resistance markers were cloned by a BmgBI and NsiI double digestion and ligation) followed by antibiotic selection, clone expansion and genotyping as above, using primers listed in **Supplementary Table 3**. The same approach was used for targeting the *AAVS1* locus and subsequent genotyping.

**Cell culture**

**hPSC generation**

AT1 iPSC line was derived from the dental pulp of a female subject, using lentivirus-derived reprogramming as previously described[^2^](#_ENREF_2). In brief, AT1 fibroblasts were cultured in hPSC-fibroblast medium (detailed below), and transduced with lentivirus containing the Yamanaka factors using a multiplicity of infection (MOI) of 10. Transduced fibroblasts were then plated onto mouse embryonic fibroblasts and iPSC clones were positively isolated using TRA-1-81 magnetic bead separation. REBL-PAT line was derived from a skin punch biopsy from a male subject, and transduced using CytoTune-iPS Sendai Reprogramming (Thermofisher #[A16517](https://www.thermofisher.com/order/catalog/product/A16517)). iPSC colonies were then isolated by manual dissection. Additionally, male HUES7 hESC[^3^](#_ENREF_3) were obtained for generation of isogenic lines. All the hPSC lines were routinely cultured in Matrigel (MT)-coated vessels in Essential 8 (E8) medium, as described below.

**hPSC culture**

All cell culture experiments were performed in a type II Biological Safety Cabinet, and cells were incubated in a humidified incubator, at 37°C and 5% CO2 (Heracell). hPSCs were routinely maintained in E8 medium (LifeTechnologies #A1517001) on 1:100 MT (Corning #356235)-coated plasticware (Nunc). Cells were passaged every 3 days by washing once with Ca^2+^/ Mg^2+^-free Phosphate Buffer Saline (PBS, Gibco #14190-094), followed by incubation with a cell dissociation solution (CDS) made of RPMI 1640 basal medium (Gibco #21875034), cell dissociation buffer (Invitrogen) #1315104) and trypsin-EDTA 0.25% (Gibco #25300054) on a 2:2:1 volume ratio, for 4 minutes at 37°C. Thereafter, CDS was quenched by washing cells with DMEM-F12 (Gibco #11320033) basal medium supplemented with 15% Knockout Serum Replacement (Gibco #10828028), 1% Non-Essential Amino Acids (NEAA, Gibco #11140050), 1% GlutaMAX (Gibco#35050061), 100 μM β-mercaptoethanol (Sigma #63689) and basic Fibroblast Growth Factor (Peprotech 100-18B, 8ng/ml), followed by centrifugation at 160xg for 4 min. Afterwards, hPSC were resuspended in E8 supplemented with 10μM Y-27632 (ROCKi, Tocris Bioscience #1254/10) and seeded into new MT-coated flasks at approximately 20000 cells/ cm^2^. Medium was changed every day. Cell lines were used between passages 20-50.

**Monolayer cardiac differentiation of hPSC**

hPSC differentiation was performed by seeding vessels at approximately 20-40 thousand cells / cm^2^, as described above. Fresh E8 was added the next day and the pre-conditioning step of hPSC was performed the day after, by adding a MT overlay (MT diluted 1:100 in StemPro™34- Serum Free Medium (SP34, Gibco #10639011, supplemented with 1ng/ml BMP4 (R&D #314-BP-050). Approximately 16h later, medium was replaced by SP34 supplemented with 8ng/ml Activin A (ActA, LifeTechnologies #PHC9564) and 10 ng/ml BMP4. 48h later, medium was changed by RPMI supplemented with B27 without insulin (-INS, LifeTechnologies #A1895601) and KY0211 (R&D #4731) and XAV939 (R&D #3748), both at 100μM. These small molecules were added again two days later, in RPMI supplemented with B27 with insulin (+INS, LifeTechnologies #0080085-SA) instead. Thereafter, medium was changed every 2-3 days by fresh RPMI+B27+INS until day 15 of differentiation, when hPSC-CM were dissociated and replated as below, and kept in RPMI+B27+INS for approximately 10 days until phenotypic assays were performed.

**hPSC-CM dissociation**

hPSC-CMs generated by the monolayer differentiation method were dissociated using a Collagenase II-based protocol, as previously described[^4^](#_ENREF_4). Briefly, cells were washed twice with Ca^2+^ and Mg^2+^-free Hank`s Balanced Salt Solution (HBSS, LifeTechnologies #14175095). Subsequently, cardiomyocytes were incubated with 200U/ml Collagenase II (Worthington #LS004176) in Ca^2+^/Mg^2+^-free HBSS, supplemented with 1mM HEPES (Sigma #H4034), 10μM Y-27632 and 30nM N-Benzyl-p-toluenesulfonamide (BTS, TCI #B3082), all diluted 1:1000, for 3.5h at 37°C at 5% CO_2_. Thereafter, dissociated cardiomyocytes were collected from the flasks and washed with RPMI supplemented with 24μg/ml Deoxyribonuclease II (DNAseII – Sigma #D8764), followed by centrifugation at 100xg for 15min. Subsequently, cells were resuspended in warm RPMI and pipetted slowly through a 100μm cell strainer (VWR # 89508-840) into a tube. Cells were then counted using an automated CEDEX HiRes counter (Roche) and centrifuged again as above. Afterwards, cardiomyocytes were resuspended in RPMI+B27+INS for seeding into Vitronectin-N (VN, Lifetech #A14700)-coated vessels, at different densities depending on the assay.

**hPSC-fibroblast culture**

Derivation of hPSC-fibroblasts was done by adapting a previous method of forced aggregation of hPSC into EB) [^5^](#_ENREF_5) and immersing them in DMEM basal medium (Gibco #11965092) supplemented with 20% FBS, 1% NEAA, 1% GlutaMAX and 100 μM β-mercaptoethanol (named FM medium). EBs were then passaged by 0.05% Trypsin-EDTA (LifeTechnologies #10462502) for 3 minutes at 37°C, vortexed and plated as single cells in MT-coated surfaces. FM medium was changed every other day and cells were passaged by incubation with Trypsin-EDTA 0.25% for 4min at 37°C, followed by quenching in FM medium and centrifugation at 300xg for 5 min. Afterwards, fibroblasts were resuspended in FM and plated in MT-coated vessels at 6000 cells / cm^2^ and fixed the day after, as described below, prior to immunostaining.

**Phenotypic assays**

**BNP assay**

BNP assay was performed as previously described[^6^](#_ENREF_6). In brief, dissociated hPSC-CMs were seeded at 120K cells/ cm^2^ in VN-coated 96 well plates (CellCarrier, Perkin Elmer). One week later, cells with incubated with 10nM Endothelin-1 (ET1) (Sigma #E7764) or 100nM-1μM Bosentan (Sigma #**SML1265)**  for approximately 15h, after which 1μg/ml Brefeldin A (Sigma #B7651) was added to the medium and incubated for another 3h, at 37°C and 5% CO_2_. Thereafter, cells were fixed and immunostained as described below. Image acquisition was performed as described below and BNP signal intensity was determined in the perinuclear region of cardiomyocytes, and divided into high, medium and low/negative according to pre-established empirical thresholds.

## Seahorse analysis of mitochondrial respiration

The Seahorse XF96 extracellular flux analyser was used to assess mitochondrial respiration, as previously described[^7^](#_ENREF_7), using the Mito Stress Kit (Agilent). Briefly, dissociated hPSC-CM were seeded into VN-coated XF96 well plates, at a density of approximately 5000 cells/mm^2^. Cardiomyocytes were cultured for one week as above and medium was exchanged for XF base medium (Agilent Technologies #102353), supplemented with 10mM glucose (Sigma #G7528), 1mM sodium pyruvate (Sigma #S8636) and 2mM L-glutamine (Life Technologies #25030-081) 1h prior to the assay. Selective inhibitors were sequentially injected during the measurements (1.5μM oligomycin, 0.4μM FCCP, 1 μM rotenone, all from Agilent Technologies), following manufacturer’s instructions. The OCR values were further normalized to the number of cells present in each well, quantified by 1:400 Hoechst33342 (Sigma) in PBS staining using fluorescence at 355 nm excitation and 460 nm emission in an automated imaging platform (CellaVista, Synentec).

**Electrophysiology measurements**

On ~d27 monolayers were washed once with PBS. Monolayers were then dissociated to single cells by adding Accutase (Sigma) and incubating at 37 °C for 10-20 minutes. After incubation the cells were pipetted gently to ensure dissociation and RPMI+B27+10%FBS was added. The dissociated cells were then transferred to a falcon tube and centrifuged at 900 rpm for 4 minutes to pellet the cells. The supernatant was removed and the cells resuspended in RPMI+B27+10% FBS. The cells were then seeded at 30,000 cells/24 well plate well onto 10mm glass coverslips that had been pre-coated with 0.1% gelatin. 24 hours later the cells were inspected for attachment and the media changed to RPMI+B27. Cells were patch clamped between 3-8 days after seeding (d30-d35). Media (RPMI+B27) was replenished every 2-3 days.

Patch clamp recording was carried out at 36.5±0.5°C using an Axopatch 200B or 700B amplifier (Axon Instruments, USA). Pipette (Intracellular) solution (**APLowEGTA**) contained (mM): 110 K-D-gluconate, 20 KCl, 10 HEPES, 0.05 EGTA, 0.5 MgCl_2_, 0.3 Na_2_-GTP, 5 Na_2_-phosphocreatine and 5 MgATP (pH 7.4 with KOH). Extracellular solution (Tyrode’s) contained (mM): 135 NaCl, 5.4 KCl, 5 HEPES, 1 MgCl_2_, 0.33 NaH_2_PO_4_, 2 CaCl_2_ and 10 Glucose (pH 7.4 with NaOH). Pipette resistance, when filled with intracellular solution, was ~2-3.5 MΩ and pipette capacitance was reduced by coating the tip with SigmaCote. Once the whole-cell configuration had been achieved action potentials were recorded in the current-clamp mode. For cells that were spontaneously firing their activity was recorded and then a trigger was applied (100pA-2nA depending on cell size and response) at a frequency of 1 Hz. For cells that were quiescent a trigger (100pA-2nA depending on cell size and response) was applied at a frequency of 1 Hz. Data was analysed using Clampfit. Action potentials were Liquid Junction Potential (LJP) corrected (LJPc). The LJP (16.7 mV) was calculated using the Clampex Junction Potential Calculator. Ten consecutive triggered action potentials were averaged, to provide a representative action potential, and these action potentials were taken after at least 10 pre-triggered APs (usually from sweeps 50-60). Electrophysiology measurements using the CellOPTIQ were performed as previously described[^8^](#_ENREF_8).

**Molecular Biology**

**RNA extraction, cDNA synthesis and RT-PCR**

Total RNA was extracted from cell pellets using the RNeasy Mini Kit (Qiagen), and reverse transcribed using SuperScript™III Reverse Transcriptase kit (Invitrogen), according to manufacturer’s instructions. RT-PCR reaction was performed using Phusion DNA polymerase (NEB) and parameters were as described above for standard PCR reaction, using exonic *MYH7* primers displayed in **Supplementary Table 3**. Subsequent PCR and gel electrophoresis were performed as above.

### **Allele-specific restriction digestion and band densitometry**

*MYH7* expression PCR was performed followed by DNA purification, as described above. Thereafter, 150 ng DNA were incubated with XhoI restriction enzyme (NEB), following manufacturers’ instructions, with undigested DNA being used as a control. DNA fragments were then loaded into a 1.2% agarose gel, run for 2h at 75V, followed by image acquisition using EtBr, as previously described. Band densitometry analysis was performed using AIDA Image Analyzer software (Raytest, GmbH), following manufacturers’ instructions.

### **Real-time qPCR**

Real-time qPCR reactions were performed via TaqMan^®^ Gene Expression Assays (Applied Biosystems) following manufacturer’s instructions. Briefly, Taqman^®^ mastermix (#4369016) including the probe of interest (*MYH7*–Hs01110632_m1, *MYH6*-Hs01101425_m1, *TNNT2*-Hs00165960_m1, *mt-ND1*- Hs02596873_s1, *mt-ND2*- Hs02596874_g1 or *ACTB*-Hs03023880_g1 was added to a MicroAmp Fast 96 well plate (#4346907). Subsequently, DNA samples (from initial 500 ng of reverse-transcribed RNA for expression studies, or 25ng from isolated DNA for mitochondrial content analysis) were added to the plate which was thereafter sealed with a film (#4311971). Amplification was performed in ABI 7500 Real-Time PCR system (Applied Biosystems). Normalisation was performed using the cardiac gene *TNNT2* for expression data, or nuclear gene *ACTB* for mitochondrial content analysis, as previously done [^9^](#_ENREF_9). The average of WT isogenic cardiomyocytes lines was used for calculating relative quantification, using the ΔΔCT method [^10^](#_ENREF_10).

### **Immunocytochemistry (ICC) and high content imaging**

Dissociated hPSC-CMs or hPSC were cultured in VN- or MT-coated 96-well plates (CellCarrier, Perkin Elmer #6005550) respectively, at approximately 50K cells/cm^2^ as described above. Cells were washed with PBS and fixed in 4% Paraformaldehyde (PFA, Sigma) at RT for 15 min. Afterwards, cells were washed in 0.1% Tween-20 (Fisher Scientific) in PBS, permeabilized with 0.1% Triton-X (Sigma) in PBS for 15 min at RT, and incubated with 4% goat serum (Sigma) in PBS (blocking solution) for 1h at RT, to prevent unspecific antibody binding. Subsequently, primary antibody incubation was performed overnight at 4°C in blocking solution, at the following dilutions: anti-OCT4-1:200 (Santa Cruz Biotechnology #SC-5279), anti-SOX2-1:200 (R&D Systems #AF2018), anti-NANOG-1:200 (R&D Systems #AF1997), anti-SSEA4-1:1000 (Milipore #MAB4304), anti-SSEA3-1:200 (Milipore #MAB1434), anti-α-actinin-1:800 (Sigma #A7811), anti-TroponinT-1:500 (Abcam #45932), anti-ProBNP4-1:500 (Abcam #13115), anti-β-MHC-1:1000 (Sigma #M8421), anti MLC-2v 1:4000 (Abcam # ab79935), anti MLC-2a 1:300 (Abcam # ab68086). Thereafter, samples were washed 3 times with 0.1% Tween-20 in PBS and incubated with Alexa Fluor secondary antibodies (Life Technologies) 1:400 in blocking solution for 1h at RT. Afterwards, cells were washed with 0.1% Tween-20 in PBS for (3x 5min), followed by nuclei and/or whole cell counterstaining with 0.5 µg/ml DAPI (Sigma #D9542) or Cell Mask (1:10000, Invitrogen #H32721) in PBS, respectively, for 30 min at RT. Samples were subsequently washed and stored at 4°C in PBS until automated image acquisition was performed in the Operetta high-content imaging system (Perkin Elmer) and analysed using Harmony high-content imaging analysis software, with the developed algorithms for determination of cardiomyocyte purity, multinucleation, BNP expression and sarcomeric disorganisation, as explored in main text and **Figures 3 and S3.**

Evaluation of sarcomeric disorganisation was performed by analysing the morphological and texture properties of cardiomyocytes immunostained for α-actinin/ cell mask/ DAPI in a PhenoLOGIC™ machine learning imaging algorithm (adapted from Perkin & Elmer). This automated method relied on sharpening the sarcomeric signal (α-actinin or cardiac troponin T) using a mathematical correction (sliding parabola) to achieve higher signal resolution (**Fig.S3H1,2**). Upon detection and identification of labelled cardiomyocyte cellular components (nuclei, cytoplasm, sarcomere, **Fig SH3-4**), sarcomeric morphology was evaluated using a STAR method assessing a set of properties including those of symmetry, threshold compactness, axial, radial and overall profile[^11^](#_ENREF_11). Textures analysis of sarcomeric signal was then performed using SER properties, Haralick features, and Gabor attributes, as measured by the software (**Fig. S3H5**). The operator has then been blinded and manually selected cardiomyocytes showing organised or disarrayed sarcomeres, in approximately 50 fields of view. Subsequently, the software compared the two classes selected and generated a model whereby parameters of training indicate a goodness of fit factor, consistent with the clustering of samples according to the morphological and texture properties measured (**Fig S3I**). Finally, the generated model was applied to all the conditions to be analysed (genotypes) in an automated manner, and the percentage of cardiomyocytes showing organised vs disarrayed sarcomeres was calculated.

**hPSC karyotyping**

hPSC were karyotyped as previously described[^12^](#_ENREF_12). In brief, exponentially growing hPSC cultures (at least 1x10^6^ cells) were treated with 100 ng/ml KaryoMAX™ Colcemid solution (Gibco #15210040) for 1h and harvested by CDS-passaging. Pelleted cells (200×g for 4 min) were resuspended in 0.6% sodium citrate (Fisher) and incubated at RT for 20 min. Cells were then centrifuged (400×g for 4 min) and fixed by resuspension in 16.7% glacial acetic acid (Fisher) in methanol (Fisher) before washing with two further changes of fixative. Chromosome spreads were prepared by dropping cells onto glass slides, which were air dried and heated to 70°C overnight. Chromosomes were G-banded by trypsin treatment and staining with Leishman’s. For each culture, at least 30 metaphase spreads were examined; full analysis involving band by band comparison between chromosome homologues was performed on three spreads and presence of gross abnormalities visually examined in 30 spreads, in accordance with ISCN Human Cytogenetic Nomenclature international guidelines.

**Flow cytometry**

### **Volumetry**

Flow volumetry analysis was performed by adapting an existing method[^13^](#_ENREF_13) to hPSC-CMs. In order to establish a calibration curve relating forward light scattered and cell size, calibration beads of known dimensions (2-14.3μm diameter, Spherotech #PPS-6K) were analysed using Astrios Cell Sorter flow cytometer (Beckman Coulter), after excluding debris and duplets/triplets. Thereafter, dissociated hPSC-CMs at day 15 of differentiation were analysed in the same manner and estimation of cardiomyocyte volume was performed using the calibration curve and formula of volume of a perfect sphere ($V= \frac{\pi}{6}\times{diameter}^{3})$. Violin plots of 25000 cells per sample were made using Stata SE14 (StataCorp LLC).

### **Quantification of mitochondrial content and ROS**

Mitochondria of dissociated hPSC-CMs were stained using 100nM MitoTracker green FM (Life Technologies #M7514). Intracellular ROS production by hPSC-CMs was investigated by staining with 2.5μM CellROX green (Life Technologies #C10444) or 5μM MitoSox Red (Life Technologies #M36008). Live cells were incubated with the dyes (made in Ca^2+/^Mg^2+^ -rich HBSS (Gibco #14025) for 30 minutes at 37°C and 5% CO_2_, followed by a PBS wash and centrifugation/ resuspension in PBS. Samples were then stored on ice before being run on Astrios flow cytometer (Beckman Coulter).

### **Data analysis**

Flow cytometry data was analysed in Kaluza software (Beckman Coulter), after removing cell debris and duplets/ triplets and using unstained samples as controls. Mean signal fluorescence intensities were used to compare mitochondrial content and ROS production in hPSC-CMs relative to wild-type (WT) isogenic controls, over 5-7 experimental replicates.

**Human Engineered Heart Tissues (hEHTs)**

### **hEHT fabrication and maintenance**

hEHTs were fabricated as previously described[^4^](#_ENREF_4)^,^ [^14^](#_ENREF_14). In brief, teflon spacers (EHT Technologies #C0002) were inserted in 2% ultra-pure agarose (Invitrogen #15510-027) solution pipetted into 24-well plates (Nunc) before gellification. Thereafter, spacers were removed and silicone racks (EHT Technologies #C0001) were placed in the aperture left by the agarose casting molds. Subsequently, freshly dissociated hPSC-CMs were resuspended in DMEM medium (Biochrom, F0415), supplemented with 10% heat-inactivated fetal calf-serum (Biochrom #S0615), 1% penicillin/ streptomycin (PEST, Gibco), 2mM L-glutamine, 2xDMEM (equalizing the hypotonic volume of fibrinogen plus thrombin), 10% MT, 0.1% Y-27632 and 5mg/ml fibrinogen (Sigma #F8630). The CM-fibrinogen mix was then quickly mixed with 3U thrombin (Sigma #T7513) and pipetted into the aperture between the silicone posts. Forming hEHTs were then incubated for 2h at 37°C and 7% CO_2_ and subsequently moved to new 24-well plates filled with DMEM medium supplemented with 10% horse serum, 10 µg/ml insulin (Sigma #I9278), 33 µg/ml aprotinin (Sigma #A1153) and 1% (v/v) PEST, termed EHT medium. Each hEHT consisted of 1 million hPSC-CMs and was fed every other day for 2-3 weeks until fully mature.

### **Analysis of contractile force**

Contractile force was analysed as previously described [^15^](#_ENREF_15). Briefly, 2-3 week-old hEHTs were immersed in modified Tyrode’s solution (120 mM NaCl, 5.4 mM KCl, 1 mM MgCl_2_, 1.8 mM CaCl_2_, 0.4 mM NaH_2_PO_4_, 22.6 mM NaHCO_3_, 5 mM glucose, 0.05 mM Na_2_EDTA, and 25 mM HEPES) pre-equilibrated overnight (37°C, 7% CO_2_, 40% O_2_), and the 24 well-plate was placed inside a transparent chamber to maintain homeostatic temperature (37°C), CO_2_ (7%) and O_2_ (40%). Automated video-optical recordings of silicone post deflection were enabled by the EHT analysis instrument (EHT Technologies #A0001) where a video camera (Basler A6021-2) placed above the chambertracked hEHT movement and a separate computer running a customized software (CMTV GmbH) determined contractile force based on the known mechanical properties of the silicone posts. When indicated, EHTs were electrically paced (2 V, 1-2.5 Hz, impulse duration 4 ms) with carbon electrodes using a Grass S88X stimulator (Astro-Med). The contraction peaks were analysed in terms of frequency (beat rate), force, and contraction (T_1_) and relaxation time (T_2_) at 80% of peak height. For omecamtiv mecarbil (OM) treatment experiments, baseline measurements were firstly recorded from electrically-paced hEHTs (1.75 Hz for AT1 lines and 1.5 Hz for REBL-PATs) in Tyrode solution at 1mM Ca^2+^, followed by treatment with 1 μM OM for 30 min and subsequent measurements. Absolute values of contractile force, T_1_ and T_2_ were reported for baseline characterization of hEHTs, and relative quantification was performed upon OM treatment by plotting changes from baseline untreated conditions.

**Confocal line scans of calcium transients**

### **hPSC-CM preparation**

Targeted GECI hPSC-CMs were differentiated as previously described, cultured in RPMI+B27+INS without phenol red (Gibco #11835030) and dissociated on day 15. hPSC-CMs were seeded at a density of 150,000 cells per well in VN-coated 35mm-diameter MatTek dishes (Nunc). CMs were assayed no earlier than day 24 and no later than day 30 of differentiation.

### **Image acquisition**

Intracellular calcium transient measurements were made using an LSM 880C confocal microscope (Carl Zeiss) in the line-scan mode, as previously described[^16^](#_ENREF_16). Briefly, CMs were located using a 40x oil objective and a longitudinal line was drawn across a single CM. Line-scan images were taken every 75 milliseconds for a total of 4000 cycles resulting in a 5 minute scan. CMs were kept at 37°C and 5% CO_2_ throughout data acquisition.

### **Data analysis**

Confocal line scan images were analysed in Fiji software (National Institute of Health). The average fluorescence intensity of each line was calculated over time to generate a confocal line-scan trace. Using the ‘multi kymograph’ Fiji plugin, a corresponding kymograph image was produced. In order to calculate beat rate and arrhythmic events, data was fed into pClamp software (Molecular Devices). Baselines were adjusted to account for photobleaching, and calcium transients were counted and analysed using the ‘event detection’ function. In order to determine abnormal DAD-like calcium transients, median peak height analysis was performed using Excel (Microsoft), wherein the median peak height for a line-scan image was calculated, and any calcium transient events that were below 75% of the peak height were considered ‘abnormal’, including those that did not return to baseline and gave a ‘double peak’.

## Transcriptomics analysis

**NanoString arrays**

Gene expression analysis was performed with nanoString nCounter Elements technology by hybridizing 50ng of RNA extracted from hPSC-CMs (as above) with a customized nanoString Gene Expression CodeSet. Analysis was done using the nCounter Sprint Profiler, following manufacturer’s instructions. The mRNA levels were normalized to five housekeeping genes (*ABCF1, CLTC, GAPDH, PGK1, and TUBB*) and expressed as fold change in mutant lines over isogenic control hPSC-CMs.

**RNA sequencing**

RNA-seq libraries were constructed using the Illumina TruSeq Stranded Total RNA sample preparation kits (Illumina, Inc., San Diego, CA), according to the manufacturers guidelines, and then sequenced on Illumina HiSeq 4000 generating 20–50 million 75 bp paired-end reads per sample. Reads were then aligned to GRCh38 human genome and transcript abundance was quantified using Salmon, as previously described[^17^](#_ENREF_17), and then imported to R. Developed models of count-based differential expression analysis of RNA sequencing data were based on the R package DeSeq2, as previously described [^18^](#_ENREF_18), using 3 biological replicates per sample.

**Statistical analysis**

Values presented in text and Figures are as mean ± standard deviation (SD), of at least three biological replicates, referred to as ‘N’, performed in triplicate (unless stated otherwise), according to Figure legends. Statistical analysis was performed by using Graph Pad Prism (v7, La Jolla, CA, USA) software, evaluated by unpaired one-way ANOVA test by using Dunnett’s post hoc test for correction of multiple comparisons (relative to respective isogenic WT lines), or by Student’s t tests to compare treated vs respective vehicle control for each genotype, or between genotypes. Differences were considered significant when *p < 0.05, **p < 0.01, ***p < 0.005 and **** p < 0.0001, color-coded according to the category compared (black asterisks indicate changes in all three categories). The numeric p values were represented whenever visually possible, but a full list is included in Supplementary Table 4.

**Supplementary Figure 1. Genotypic characterization of genome-edited hPSC. A)** CRISPR/Cas9 gene targeting strategy used to edit *MYH7* locus relied on FRT-mediated excision of antibiotic resistance cassette that constituted the homology targeting vector containing the *C9123T* nucleotide change. **B)** Simplified schematic of the *MYH7* gene post gene targeting as well as the PCRs used to genotype picked colonies (numbered) – at least one of the alleles was expected to have a resistance cassette in positive colonies, which was removed upon **C)** Flippase-mediated recombination. **D)** DNA electrophoresis gels showing the results of the allele-specific PCR amplification of: **1**) Left homology arm (cassette allele); **2**) right homology arm (cassette allele); **3**) Non-cassette allele; **4**) both alleles. **E)** PCR-genotyping of positive colonies upon selection cassette excision: 5) Flipped colonies have a smaller *MYH7*-PCR product (~0.6Kb) than the unflipped ones (~2.8Kb); 6) colonies lose resistance cassette DNA upon cassette excision. **F)** Sequencing of the off-target locus *MYH6* indicates disruption of the reading frame (frameshift mutation) in one gene-edited hPSC colony (likely due to NHEJ), homozygous for R453C-βMHC change. L/RA, left/right homology arm; MW, Molecular weight marker; NTC, non template control; HDR, homologous directed recombination; NHEJ, non-homologous end-joining.

**Supplementary Figure 2. Characterization of gene-edited hPSC and hPSC-CMs. A)** Representative fluorescent micrographs of genome-edited hPSC lines indicate expression of pluripotency markers OCT4, SOX2, NANOG, SSEA4 and SSEA3 (green boxes), in comparison to secondary antibody only and/or fibroblast controls (red boxes). Scale bar=200 μm. **B)** Representative karyogram of genetically stable hPSC lines. **C)** Monolayer cardiac differentiation protocol overview: a matrigel overlay was applied to E8-cultured hPSCs, followed by Wnt pathway activation (Stage 2) and inhibition (Stage 3), resulting in beating sheets of hPSC-CMs 8-12 days after the differentiation started. **D)** Representative fluorescence micrographs of AT1-CMs co-stained for β-MHC, cardiac troponin T and DAPI, confirming protein expression of the *MYH7* gene in cardiomyocytes, with the exception of the *MYH7*-knockout line. Scale bar = 100 µm.

**Supplementary Figure 3: Subtype characterisation of hPSC-CMs. A)** hPSC-CMs predominantly expressed ventricular marker MLC2v, showing MLC2v/α-actinin and MLC2v/ML2a ratios >90%. (Bar=20μm- top row, 100μm – bottom row). **B)** Functional evaluation of subtype specification by patch clamp of single cells or CellOPTIQ-based optical imaging of synchronous monolayers. In **B)**, representative traces are shown for the different cardiac subtypes. Two metrics of cardiac subtype were applied: **C)** APD_90_/APD_50_ and **D)** (APD_80_-APD_70_)/APD_40_-APD_30_). F test to compare variances showed data from single cells was significantly more variable than synchronous monolayers. Irrespective of the method used for experimentation or analysis, most cells were of ventricular-like subtype. Patch was 49 data points across 3 biological replicates; CellOPTIQ was 80 data points across 4 biological replicates.

**Supplementary Figure 4. High content imaging analysis pipeline. A)** Representative fluorescent micrographs of BNP/cTnT/DAPI-imunostained hPSC-CMs upon Bosentan (100nM) and ET-1 (10 nM) treatment (**B**) (Scale bars = 100 μm). Quantification of BNP expression by the automated algorithm shows increased proportion of cardiomyocytes expressing the hypertrophic marker BNP in gene-edited lines in comparison to their respective isogenic controls (**C**), which is rescued by Bosentan treatment in REBL-PAT-CMs, but not in HUES7-CMs (**D**), and maximised in all lines by ET-1 treatment (**E**), N=6. **F**) Multinucleation analysis of gene-edited REBL-PAT and **G**) HUES7 cardiomyocytes show increased proportion of bi- and multinucleated cells in heterozygous R453C-βMHC hPSC-CMs relative to their respective isogenic control, corroborating the phenotype observed in the AT1 cell line (N=8). **H**) Algorithm to quantify of sarcomeric disarray: **1)** input image of imunostained hPSC-CMs for sarcomeric marker α-actinin using cell mask and DAPI as counterstains; **2)** sharpen the signal derived from the sarcomeric staining to enable higher resolution, using sliding parabola correction; **3)** find nuclei of stained cells; **4)** find cytoplasm using Cell mask counterstain channel; **5)** calculate morphology and texture properties of cardiomyocytes in the cytoplasm, using sharpened α-actinin signal. **I)** Parameters of software-training indicate goodness of fit factor as well as the main texture/morphological properties that differ between organised vs disarrayed sarcomeres. Data plotted as mean +/- SD. One-way ANOVA tests with Dunnett’s correction for multiple comparison were performed to compare mutant lines with their isogenic control (vehicle or untreated conditions). Student’s t tests were used to compare treated vs respective vehicle control for each genotype (*p<0.05; **p<0.01; ***p<0.005; ****p<0.0001), color-coded according to the category compared (black asterisks indicate changes in all three categories). Absolute numbers in Supplementary Table 4.

**Supplementary Figure 5. In silico analysis and modelling of βMHC interactions**. **A-C)** The R453C mutation is predicted to interfere with the S1(head - blue)-S2(tail - yellow) hydrogen bonding. Shown in a is also a model of MyBP-C bound to the free head (orange)[^19^](#_ENREF_19). **D,E)** The R453 residue is predicted to be close to bound βMHC and is seen to form interactions with the C1 domain in molecular dynamics simulations (CHARMM C36b2[^20^](#_ENREF_20)). R453 is distal to the predicted actin binding interface (**F**) and the nucleotide binding site in S1 (**G**) and is therefore not predicted to directly disrupt their interaction[^21^](#_ENREF_21). Figures produced using PyMOL.

**Supplementary Figure 6. Cardiac bioenergetics analysis of REBL-PAT HCM lines. A)** Mitochondrial respiration profile of REBL-PAT R453C-βMHC hPSC-CMs using the Seahorse platform showed similar **B)** basal respiration and **D)** ATP production, with **C)** increased maximal respiration rates in edited lines relative to the isogenic control (OCR-oxygen consumption rate, N=5). **E)** qPCR analysis of the ratio of mitochondrial (ND1-2)/nuclear (β-actin) DNA showed a decreasing trend of mitochondrial content in gene edited lines (N=3). Representative histograms of gene-edited hPSC-CMs labelled with **F)** Mitotracker, **H)** Cell ROX and **B)** MitoSOX obtained by flow cytometry. Quantification of signal intensity from flow data did not show any differences in mitochondrial content (**G**) and production of ROS species (**I-K**), with the exception of the CellROX labelled-homozygous line, N=7. Data plotted as mean +/- SD. One-way ANOVA tests with Dunnett’s correction for multiple comparison were performed to compare mutant lines with their isogenic control (P values in graphs).

**Supplementary Figure 7. Characterization of R-GECO-edited R453C-βMHC lines**. **A)** Schematics of *AAVS1* gene post gene-targeting, as well as the PCRs used to genotype picked colonies (numbered). **B)** DNA electrophoresis gels showing the results of the PCR amplification of: **1)** Left homology arm; **2)** right homology arm. **C)** Confirmation of correct *RGECO1-AAVS1* gene editing by sequencing left/right arm PCR products. **D)** Split channel view of α-actinin/ DAPI immunostained cells highlight expression of RGECO1 in gene-edited REBL-PAT-cardiomyocytes (scale bar = 50µm)**. e)** Representative traces of ranolazine treated hPSC-CMs showing a reduction in the number of abnormal events (quantified in Fig. 5I,J).

**Supplementary Figure 8 - Contractile force analysis in REBL-PAT-EHTs. A)** Histological analysis of an EHT section reveals highly aligned cardiomyocytes in the fibrin gel (scale bar = 20 µm) **B)** Spontaneous beat rate of REBL-PAT EHTs showed statistically significant increase in mutant lines relative to the WT control (N=6). **C)** Average contraction peaks of paced *MYH7*-mutant REBL-PAT-EHTs reveal a hypo-contractile (quantified in (**D**) and marginally-negative clinotropic phenotypes (**E**) accompanied by a slight increase in the relaxation time (**F**), in comparison to the healthy isogenic control (N=6). **G)** Average contraction peaks of REBL-PAT EHTs in baseline conditions (black line) and upon OM treatment (purple line) in the different genotypes. **H)** OM treatment caused a decrease in the contraction force in all lines, which was more pronounced in the R453C-βMHC mutant lines relative to the WT control. **I)** Mutant lines had a less pronounced increase in the contraction time (T1) upon OM treatment, in comparison to healthy WT line. **J)** OM treatment resulted in a decrease in the relaxation time in the WT line and no significant differences in mutant lines relative to untreated conditions. N=3, in differences upon treatment from baseline, between mutant lines and WT control. **K)** Unlike AT1-EHTs, *MYH7*-mutant REBL-PAT-EHTs did not show a more pronounced negative force-frequency relationship than the WT control (N=6, comparing paced and respective unpaced baseline). Data plotted as mean +/- SD. One-way ANOVA tests with Dunnett’s correction for multiple comparison were performed to compare mutant lines with their isogenic control). Student’s t tests were used to compare between *MYH7*-mutant genotypes (P values in figure).

**Supplemental Figure 9. Details on transcriptomics analysis. A)** PCA analysis highlights cell culture format and origin of hPSC as the main factors explaining the observed changes in gene expression. **B**) MA plots of RNA-sequencing models developed showing differentially expressed genes (290 at FDR<0.1) between healthy (WT) and diseased (*MYH7*-mutant) conditions, and **C**) when including the cell line as a correcting factor (N=3). **D**) expression changes of sarcomeric genes between heterozygous (yellow bars) and homozygous (red bars) relative to the wild-type controls indicates higher magnitudes in the latter. **E**) qPCR analysis of *MYH7/MYH6* expression ratios in gene-edited HUES7-CM lines normalised to WT controls, in 2D cultures (N=4). **F)** Fold changes in the expression of genes involved in archetypal HCM pathways in the *MYH7*-mutant AT1 cardiomyocyte lines relative to the WT isogenic control, in 2D cultures, and **G)** 3D-EHTs, with fold SD reported per each gene. Data plotted as mean +/- SD. One-way ANOVA tests with Dunnett’s correction for multiple comparison were performed to compare mutant lines with their isogenic control (P values in figure).

**Supplementary Table Legends**

**Supplementary Table 1** – guide RNA sequences used for *MYH7* targeting as well as predicted off-targets.

**Supplementary Table 2** – guide RNA sequences used for AAVS1 targeting as well as predicted off-targets.

**Supplementary Table 3** – Primers used and respective reactions/ products

**Supplementary Table 4** – P values for all data points that showed significance

1. Ran FA, Hsu PD, Wright J, Agarwala V, Scott DA, Zhang F. Genome engineering using the CRISPR-Cas9 system. Nat Protocols 2013;**8**(11):2281-2308.

2. Dick E, Matsa E, Young LE, Darling D, Denning C. Faster generation of hiPSCs by coupling high-titer lentivirus and column-based positive selection. Nat Protocols 2011;**6**(6):701-714.

3. Cowan CA, Klimanskaya I, McMahon J, Atienza J, Witmyer J, Zucker JP, Wang S, Morton CC, McMahon AP, Powers D, Melton DA. Derivation of Embryonic Stem-Cell Lines from Human Blastocysts. N Engl J Med 2004;**350**(13):1353-1356.

4. Breckwoldt K, Letuffe-Breniere D, Mannhardt I, Schulze T, Ulmer B, Werner T, Benzin A, Klampe B, Reinsch MC, Laufer S, Shibamiya A, Prondzynski M, Mearini G, Schade D, Fuchs S, Neuber C, Kramer E, Saleem U, Schulze ML, Rodriguez ML, Eschenhagen T, Hansen A. Differentiation of cardiomyocytes and generation of human engineered heart tissue. Nat Protocols 2017;**12**(6):1177-1197.

5. Burridge PW, Anderson D, Priddle H, Barbadillo Muñoz MD, Chamberlain S, Allegrucci C, Young LE, Denning C. Improved Human Embryonic Stem Cell Embryoid Body Homogeneity and Cardiomyocyte Differentiation from a Novel V-96 Plate Aggregation System Highlights Interline Variability. Stem Cells 2007;**25**(4):929-938.

6. Carlson C, Koonce C, Aoyama N, Einhorn S, Fiene S, Thompson A, Swanson B, Anson B, Kattman S. Phenotypic Screening with Human iPS Cell–Derived Cardiomyocytes. J Biomol Screen 2013;**18**(10):1203-1211.

7. Yang X, Rodriguez M, Pabon L, Fischer KA, Reinecke H, Regnier M, Sniadecki NJ, Ruohola-Baker H, Murry CE. Tri-iodo-l-thyronine promotes the maturation of human cardiomyocytes-derived from induced pluripotent stem cells. J Mol Cell Cardiol 2014;**72**:296-304.

8. Duncan G, Firth K, George V, Hoang MD, Staniforth A, Smith G, Denning C. Drug-Mediated Shortening of Action Potentials in LQTS2 Human Induced Pluripotent Stem Cell-Derived Cardiomyocytes. Stem Cells Dev 2017;**26**(23):1695-1705.

9. Burkart AM, Tan K, Warren L, Iovino S, Hughes KJ, Kahn CR, Patti M-E. Insulin Resistance in Human iPS Cells Reduces Mitochondrial Size and Function. Sci Rep 2016;**6**:22788.

10. Schmittgen TD, Livak KJ. Analyzing real-time PCR data by the comparative CT method. Nat Protocols 2008;**3**(6):1101-1108.

11. Denning C, Borgdorff V, Crutchley J, Firth KSA, George V, Kalra S, Kondrashov A, Hoang MD, Mosqueira D, Patel A, Prodanov L, Rajamohan D, Skarnes WC, Smith JGW, Young LE. Cardiomyocytes from human pluripotent stem cells: From laboratory curiosity to industrial biomedical platform. Biochim Biophys Acta 2016;**1863**(7):1728-1748.

12. Priddle H, Allegrucci C, Burridge P, Munoz M, Smith NM, Devlin L, Sjoblom C, Chamberlain S, Watson S, Young LE, Denning C. Derivation and characterisation of the human embryonic stem cell lines, NOTT1 and NOTT2. In Vitro Cell Dev Biol Anim 2010;**46**(3):367-375.

13. Tzur A, Moore JK, Jorgensen P, Shapiro HM, Kirschner MW. Optimizing Optical Flow Cytometry for Cell Volume-Based Sorting and Analysis. PLoS One 2011;**6**(1):e16053.

14. Schaaf S, Eder A, Vollert I, Stöhr A, Hansen A, Eschenhagen T. Generation of Strip-Format Fibrin-Based Engineered Heart Tissue (EHT). In: Radisic M, Black Iii LD, eds. *Cardiac Tissue Engineering: Methods and Protocols*. New York, NY: Springer New York; 2014, p 121-129.

15. Mannhardt I, Breckwoldt K, Letuffe-Brenière D, Schaaf S, Schulz H, Neuber C, Benzin A, Werner T, Eder A, Schulze T, Klampe B, Christ T, Hirt Marc N, Huebner N, Moretti A, Eschenhagen T, Hansen A. Human Engineered Heart Tissue: Analysis of Contractile Force. Stem Cell Reports 2016;**7**(1):29-42.

16. Yazawa M, Hsueh B, Jia X, Pasca AM, Bernstein JA, Hallmayer J, Dolmetsch RE. Using induced pluripotent stem cells to investigate cardiac phenotypes in Timothy syndrome. Nature 2011;**471**(7337):230-234.

17. Patro R, Duggal G, Love MI, Irizarry RA, Kingsford C. Salmon provides fast and bias-aware quantification of transcript expression. Nature Methods 2017;**14**:417.

18. Anders S, McCarthy DJ, Chen Y, Okoniewski M, Smyth GK, Huber W, Robinson MD. Count-based differential expression analysis of RNA sequencing data using R and Bioconductor. Nat Protoc 2013;**8**:1765.

19. Nag S, Trivedi DV, Sarkar SS, Adhikari AS, Sunitha MS, Sutton S, Ruppel KM, Spudich JA. The myosin mesa and the basis of hypercontractility caused by hypertrophic cardiomyopathy mutations. Nature Structural &Amp; Molecular Biology 2017;**24**:525.

20. Bloemink M, Deacon J, Langer S, Vera C, Combs A, Leinwand L, Geeves MA. The Hypertrophic Cardiomyopathy Myosin Mutation R453C Alters ATP Binding and Hydrolysis of Human Cardiac β-Myosin. J Biol Chem 2014;**289**(8):5158-5167.

21. Lorenz M, Holmes KC. The actin-myosin interface. Proceedings of the National Academy of Sciences 2010;**107**(28):12529-12534.
