# Supplementary figures and images for: CRISPR/Cas9 editing in human pluripotent stem cell-cardiomyocytes highlights arrhythmias, hypocontractility, and energy depletion as potential therapeutic targets for hypertrophic cardiomyopathy
Source: Eur Heart J. 2018 May 8;39(43):3879–92. doi: 10.1093/eurheartj/ehy249 (PMC6234851; doi:10.1093/eurheartj/ehy249)

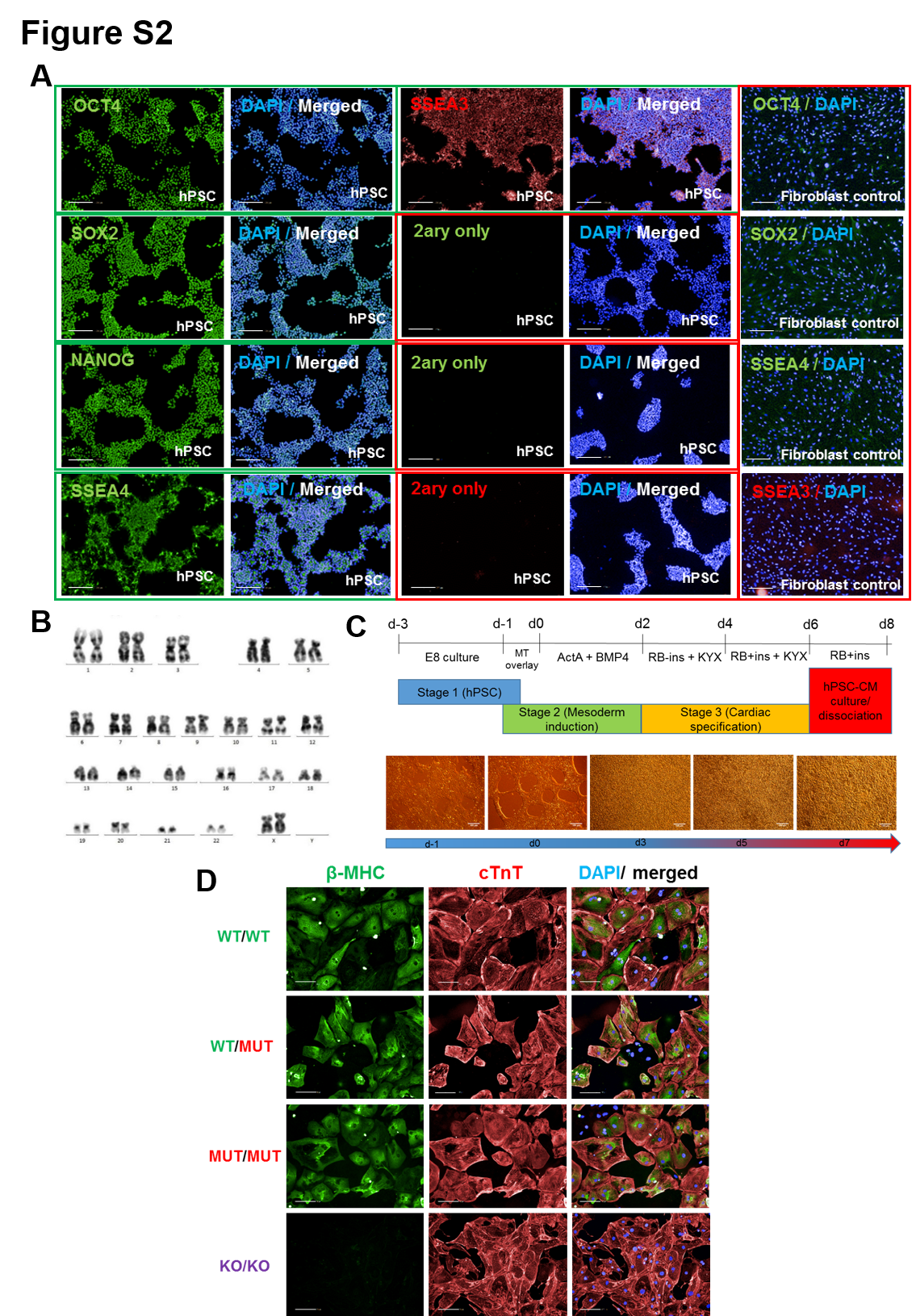

Supplement: Supplementary Data [file ehy249_supp.zip › ehy249-suppl_data/ehy249_Figure_S2.tif]

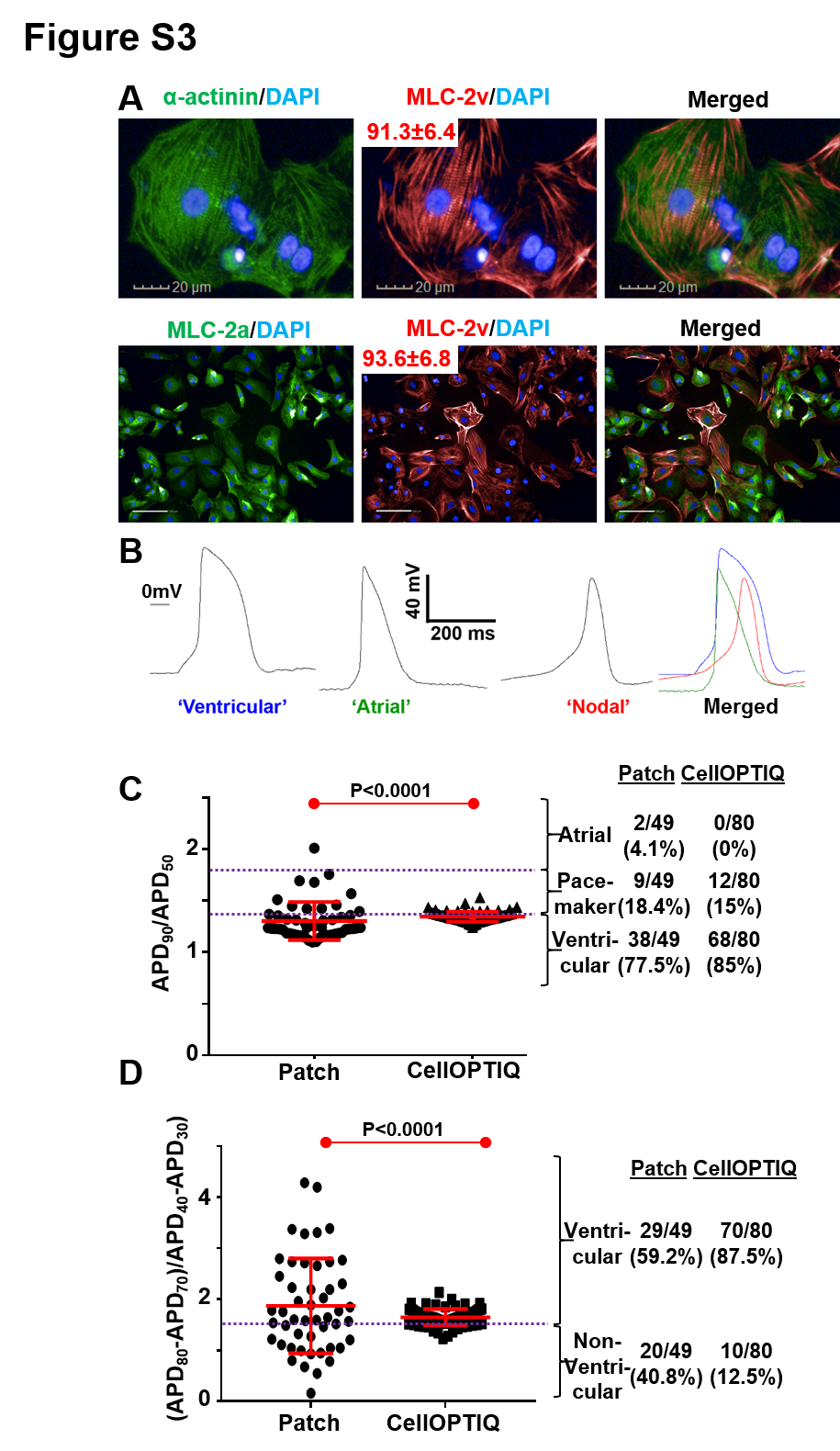

Supplement: Supplementary Data [file ehy249_supp.zip › ehy249-suppl_data/ehy249_Figure_S3.tif]

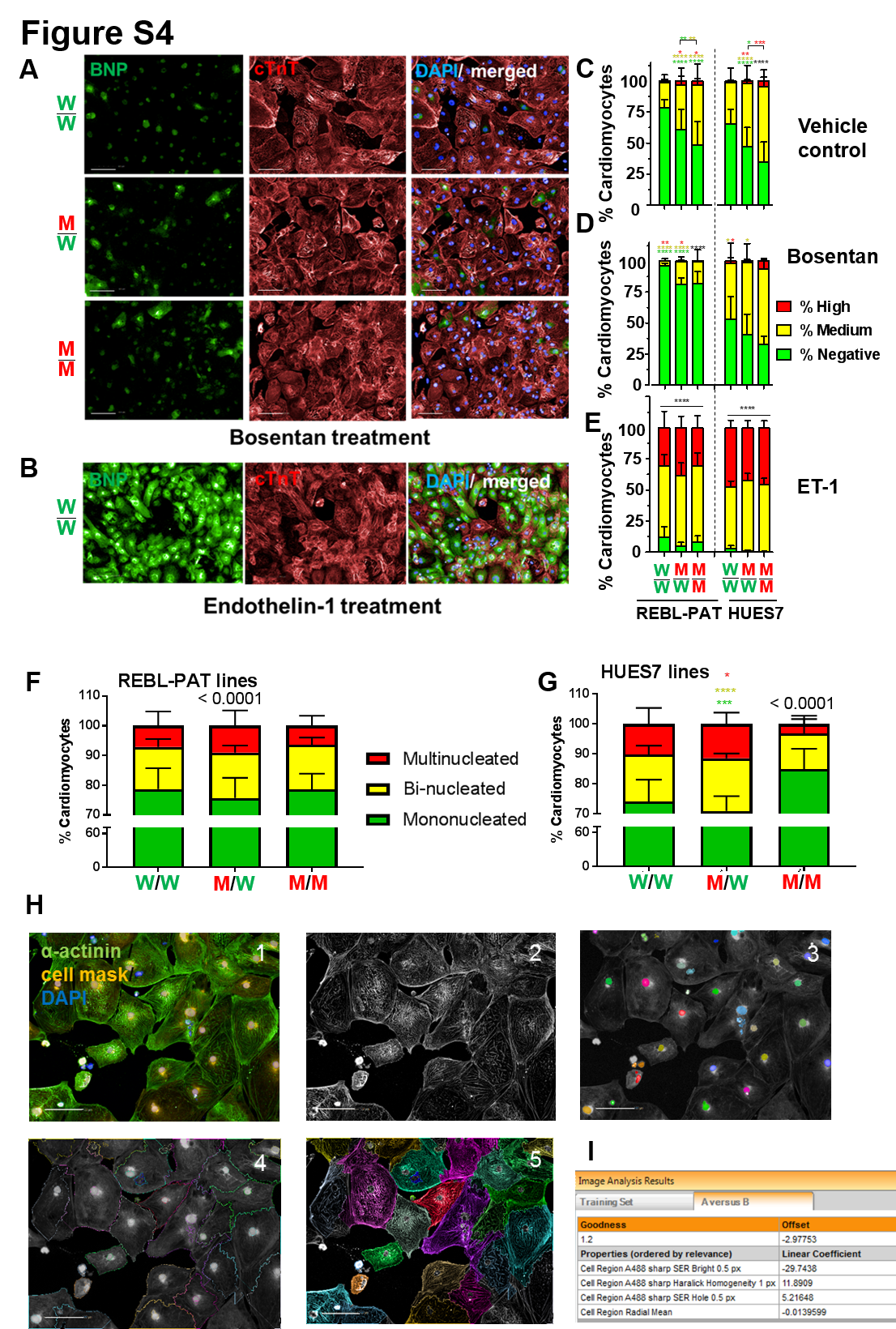

Supplement: Supplementary Data [file ehy249_supp.zip › ehy249-suppl_data/ehy249_Figure_S4.tif]

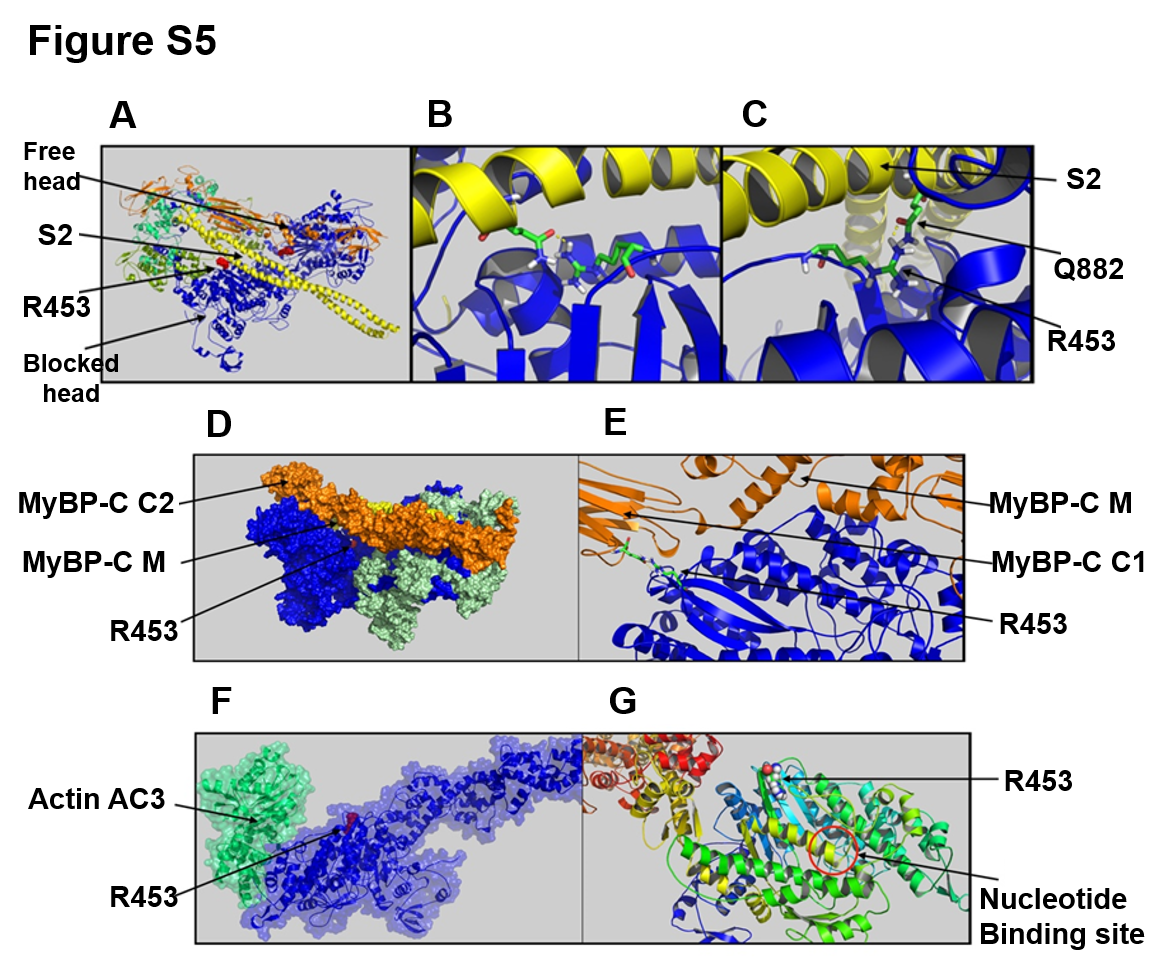

Supplement: Supplementary Data [file ehy249_supp.zip › ehy249-suppl_data/ehy249_Figure_S5.tif]

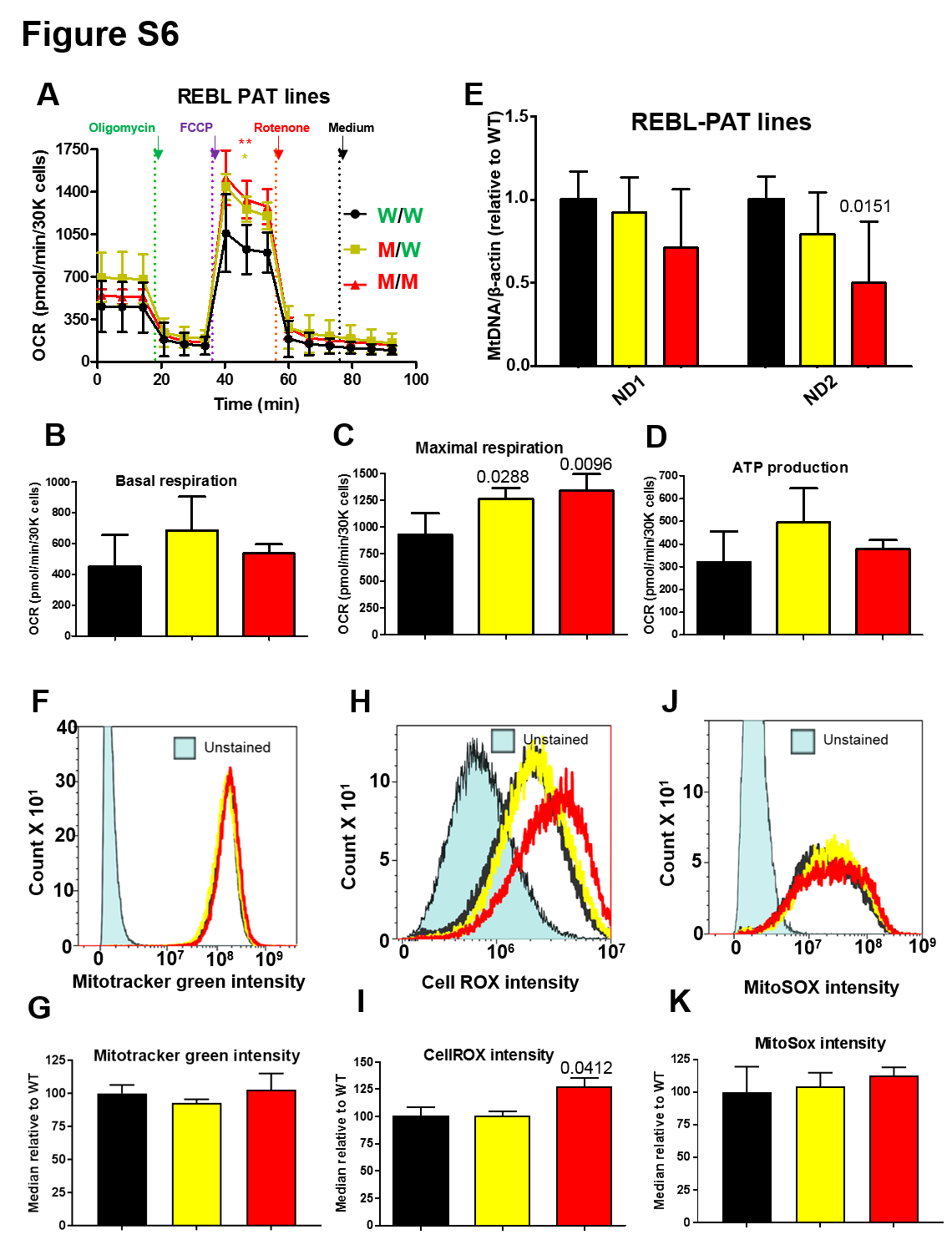

Supplement: Supplementary Data [file ehy249_supp.zip › ehy249-suppl_data/ehy249_Figure_S6.tif]

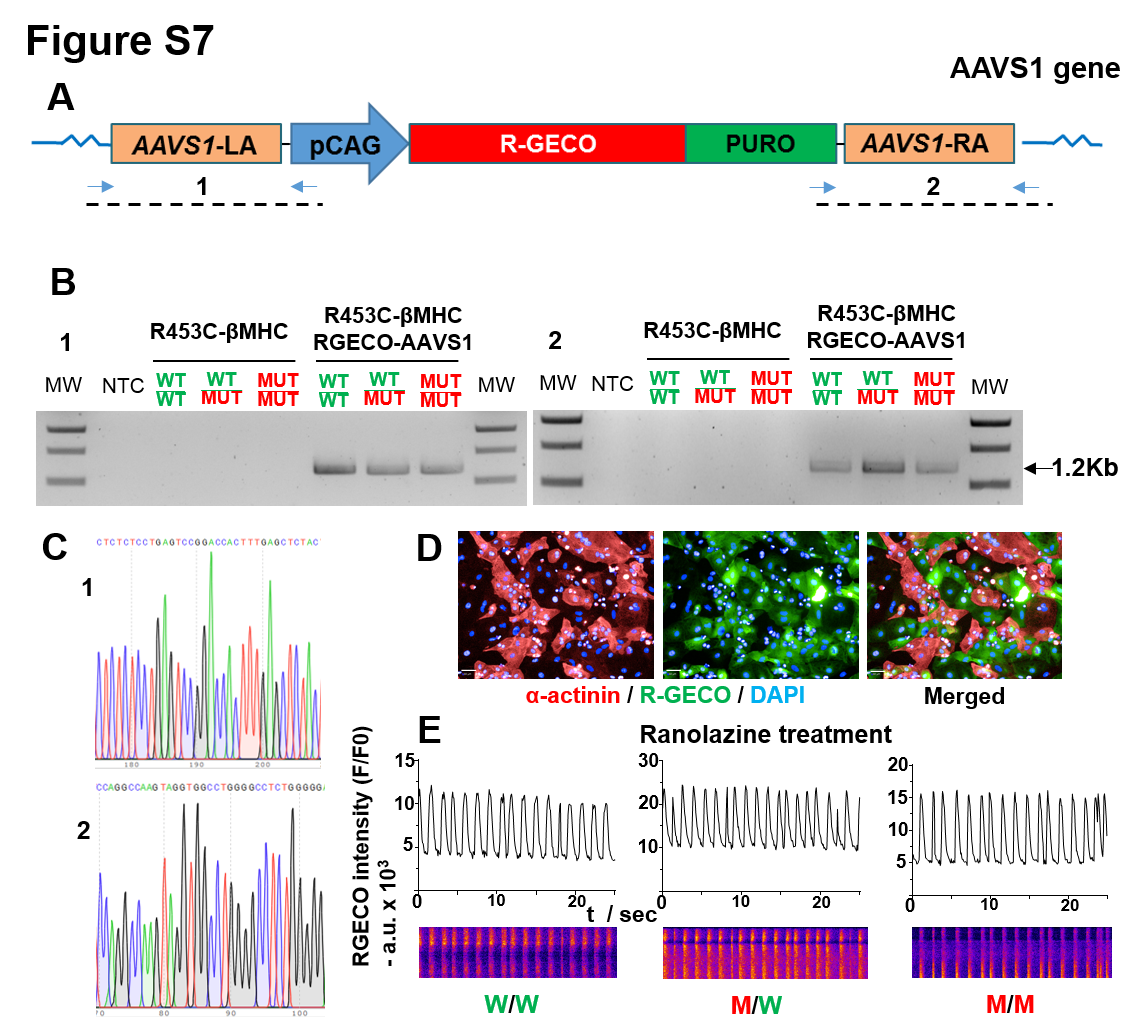

Supplement: Supplementary Data [file ehy249_supp.zip › ehy249-suppl_data/ehy249_Figure_S7.tif]

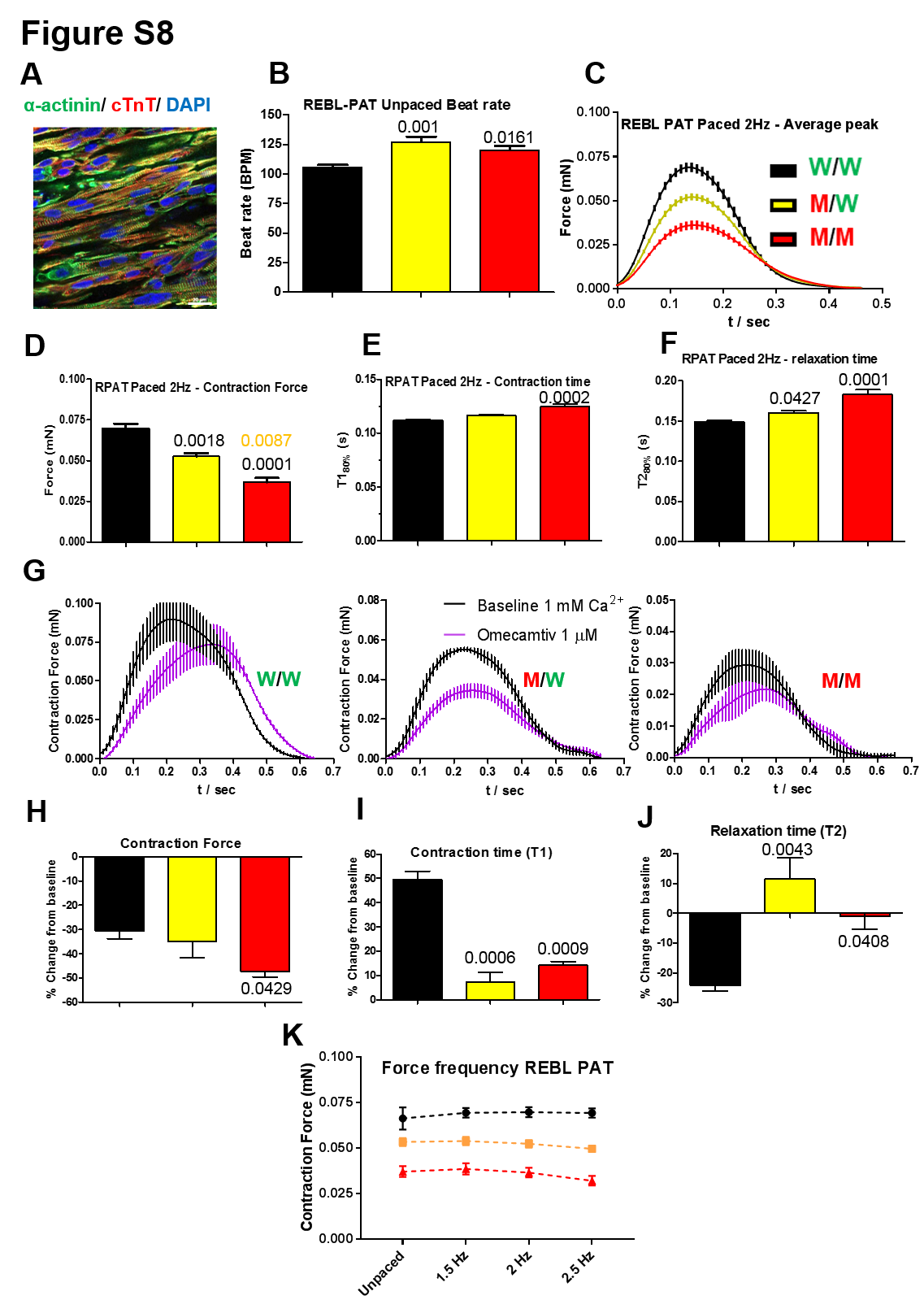

Supplement: Supplementary Data [file ehy249_supp.zip › ehy249-suppl_data/ehy249_Figure_S8.tif]

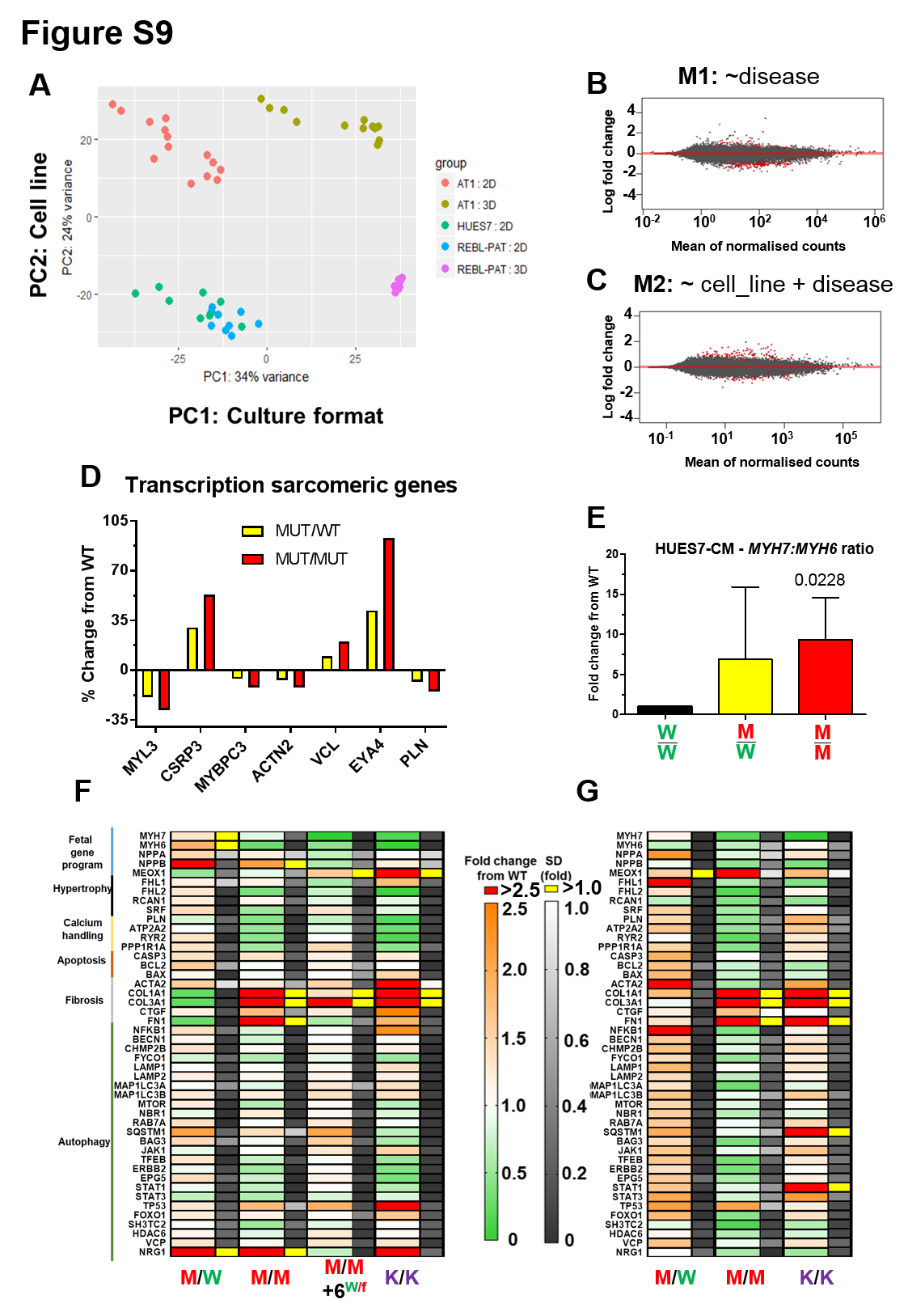

Supplement: Supplementary Data [file ehy249_supp.zip › ehy249-suppl_data/ehy249_Figure_S9.tif]

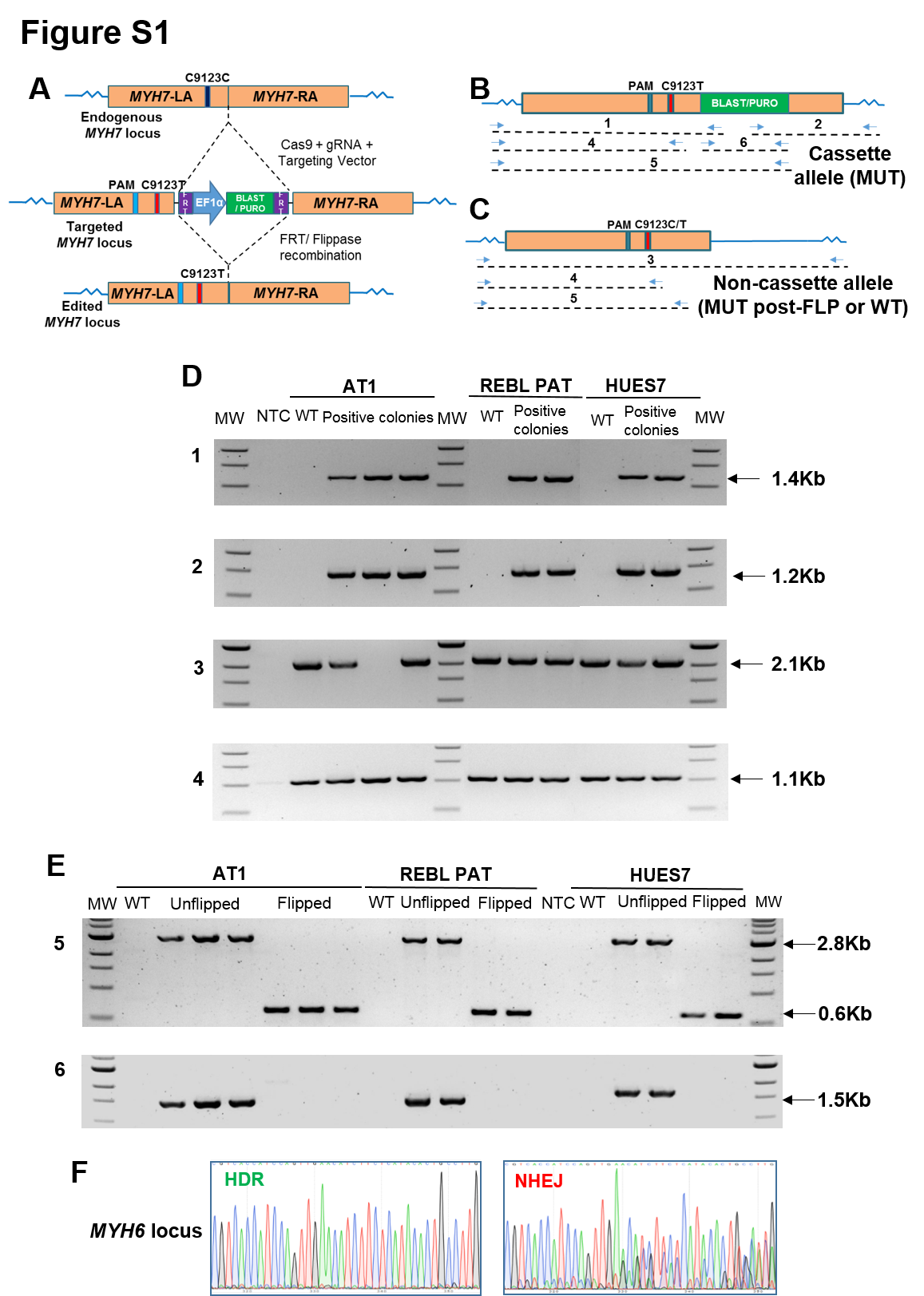

Supplement: Supplementary Data [file ehy249_supp.zip › ehy249-suppl_data/ehy249_Figure_S1.tif]
